# Enhancing BeiDou/GNSS integrity with minmax optimization

**DOI:** 10.1093/pnasnexus/pgaf366

**Published:** 2025-11-14

**Authors:** Jingsong Qiu, Ci Chen, Siyu Lei, Yi Lyu, Shengli Xie

**Affiliations:** School of Automation, Guangdong University of Technology, Guangzhou 510006, China; Guangdong Provincial Key Laboratory of Intelligent Systems and Optimization Integration, Guangzhou 510006, China; School of Automation, Guangdong University of Technology, Guangzhou 510006, China; Key Laboratory of Intelligent Information Processing and System Integration of IoT, Ministry of Education, Guangzhou 510006, China; School of Automation, Guangdong University of Technology, Guangzhou 510006, China; 111 Center for Intelligent Batch Manufacturing Based on IoT Technology, Guangzhou 510006, China; School of Automation, Guangdong University of Technology, Guangzhou 510006, China; School of Computer, University of Electronic Science and Technology of China Zhongshan Institute, Zhongshan 528400, China; School of Automation, Guangdong University of Technology, Guangzhou 510006, China; Guangdong-HongKong-Macao Joint Laboratory for Smart Discrete Manufacturing, Guangzhou 510006, China

**Keywords:** GNSS, ARAIM, vertical protection level, maximum monitoring order, minmax optimization

## Abstract

In navigation scenarios, integrity is a crucial metric for evaluating system availability, with improvements in integrity having implications for transportation, security, and surveillance. This article proposes a novel integrity monitoring strategy, termed the optimal advanced receiver autonomous integrity monitoring (Optimal-ARAIM), which is designed to optimize the vertical protection level (VPL) in the context of BeiDou/global navigation satellite systems (GNSS). Optimal-ARAIM employs a minimax estimator to minimize VPL by adjusting the full-set solution, optimally allocating integrity and continuity risks. To mitigate the combinatorial explosion caused by multiple heterogeneous satellite faults, we introduce a maximum monitoring order mechanism. All worst-case fault scenarios are formulated as a minimax optimization problem, which is approximated by a convex optimization to ensure the global convergence of VPL. To evaluate the performance of Optimal-ARAIM, we utilize BeiDou observation data for validation and almanac data for predictive analysis. The results indicate that the average VPL is consistently maintained below 8 m across five selected stations in the Asian region when using observation data. Additionally, VPL distributions predicted using BeiDou almanac data are predominantly below 10 m. Further validation using GNSS almanac data demonstrates that the proposed method achieves a global availability coverage rate exceeding 93%, meeting the CAT-I standard. These findings confirm that the proposed Optimal-ARAIM effectively reduces the VPL for BeiDou/GNSS, ensuring that navigation operations can be conducted with high robustness and reliability.

Significance Statement Innovative navigation solutions are essential for unlocking the full potential of the low-altitude economy, ensuring that it remains a dynamic and thriving sector in the years to come. A novel integrity monitoring strategy is proposed for BeiDou/global navigation satellite systems to optimize the vertical protection level. It employs a precise penalty function to transform the minmax estimator and utilizes a minimization upper bound algorithm to find the optimal solution iteratively. This advancement surpasses the stringent navigational standards required for modern airspace and paves the way for broader applications, including more resilient operations in densely populated airspaces, enhanced precision in approach and landing procedures, and potential integration into autonomous flight systems, further solidifying its pivotal role in the future of aviation navigation.

## Introduction

The initiative to fully integrate general aviation equipment (GAE) into everyday life and professional environments is set to revolutionize the low-altitude economy (LAE). In applications where lives are at stake, the integrity and control of navigation systems become mission-critical (1, 2). At the heart of reliable navigation solutions lies integrity, particularly for global navigation satellite system (GNSS)-based platforms like the BeiDou navigation satellite system (BeiDou), which has undergone advancements in recent years (3). Ensuring high integrity is essential for the safe operation of BeiDou/GNSS applications (4). As a result, the development and enhancement of integrity monitoring (IM) have become priorities in the LAE domain. For GAE applications, the capability for effective vertical guidance is vital, as it ensures that even in the face of potential system failures, the navigation system maintains positioning accuracy within the vertical protection level (VPL) (5, 6). The VPL represents the maximum vertical positioning error that a system considers possible at a given confidence level. A smaller VPL indicates higher confidence from the navigation system that the current vertical positioning error is close to the true value, thus enhancing the overall integrity and availability of the system.

The WG-C report outlines ambitious goals for IM, aiming to achieve global LPV-200 performance in dual-frequency, multiconstellation (DFMC) scenarios. The requirements for LPV-200 (Localizer Performance with Vertical guidance available down to 200 ft) are stringent: vertical accuracy of 1.86 m, a vertical alarm limit (VAL) of 35 m, and an effective monitoring threshold (EMT) of 15 m (7). In civil aviation, precision approaches must meet the rigorous standards of category I (CAT-I), which demand an even stricter VAL of 15 m. This study leverages LPV-200 as a benchmark to evaluate whether GAE-based IM methods can rise to the challenge of meeting CAT-I standards, ensuring safety and reliability in critical flight operations. For certain critical operations, such as GAE autolanding and urban canyon flight, navigation systems demand a smaller VPL at an even higher confidence level. This signifies that the system possesses a superior level of integrity support capability, which is crucial for GAE systems to meet more stringent certification standards. In essence, the navigation system must achieve both precision and assured reliability in safety-critical scenarios, thereby providing tighter error bounds to ensure safety and compliance with rigorous aviation requirements. Receiver autonomous integrity monitoring (RAIM) is the widely used method in aerial navigation, serving as an indicator of navigation systems. Initially implemented by the global positioning system (GPS) (8), RAIM verifies positioning solutions through consistency checks (9). To enhance the IM in DFMC scenarios, advanced RAIM (ARAIM) has been developed (10). ARAIM consists of three segments: the space segment for broadcasting satellite ranging information, the ground segment, which broadcasts integrity support messages (ISM), and the user segment responsible for calculating the system’s PL (11). This deployment underscores the need for BeiDou to deliver high-precision and high-integrity positioning.

The user segment of ARAIM is the subject of the implementation of the IM algorithm, which is the main focus of the study. The mainstream ARAIM method is based on multiple hypothesis solution separation (MHSS). In the context of ARAIM, the establishment of multiple hypotheses serves a crucial purpose: it enables the system to detect potential threats arising from various fault modes, including heterogeneous faults such as single or multiple satellite malfunctions. Under this multihypothesis framework, the PL takes on a more comprehensive meaning. It characterizes both the upper bound on positioning errors and the worst-case scenario under the constraints of each specific fault hypothesis. This means that for every considered failure scenario, ARAIM calculates a PL that accounts for the maximum possible positioning error if that particular fault were to occur. This rigorous approach is fundamental to ensuring the high integrity and reliability required for safety-critical navigation applications. Incorporating more constellation and satellite information for integrity monitoring can enhance the algorithm’s performance, but it also increases the number of fault hypotheses, reducing efficiency (12). To address this, fault grouping technology based on common characteristics, such as distribution and occurrence probability, was considered for improving algorithm efficiency (13). Although fault grouping technology has been validated on GPS and Galileo systems (14), its application to the BeiDou system has been limited (15). Therefore, this article explores fault grouping technology and conducts verification using BeiDou data.

In RAIM applications, there is also a more robust approach of positioning domain monitoring—DIA (16). First, observation data are detected to identify potential faults or anomalies; subsequently, fault identification is performed to estimate their failure type and magnitude; finally, adaptive adjustments are executed based on the identified fault characteristics to maintain the consistency and integrity of the positioning solution. Common DIA metrics include the minimum detectable bias (MDB) and the largest probability of containment (LPC) (17), which respectively measure fault detection sensitivity and the confidence of keeping errors within the safety domain in cases of undetected faults. In contrast, the RAIM and ARAIM frameworks focus on directly calculating the protection level—ensuring the tightness of the alert limit given integrity risk and continuity budget (18). Although both aim to enhance system robustness, DIA emphasizes real-time threshold adjustment and tolerance management based on detection–identification–adaptation, while ARAIM directly defines the upper bound of position error through multihypothesis enumeration and risk allocation. A quantitative connection between the two can be established by converting MDB to an equivalent protection level or by mapping the protection level to an equivalent detection threshold, but such system comparisons are beyond the scope of this work and will be explored in future research.

In ARAIM practice, the system needs to provide reliable positioning solutions within specified integrity risk and continuity risk parameters. Integrity risk occurs when the positioning error exceeds the alarm limit, yet the system continues to consider the location solution as valid, leading to a probability of hazardously misleading information ($P_{\mathrm{HMI}}$) (19). Continuity risk, on the other hand, refers to the system mistakenly identifying a fault with the location solution and issuing a warning (false alarm, $P_{\mathrm{fa}}$). In MHSS-ARAIM, risk requirements need to be allocated under different fault hypotheses based on $P_{\mathrm{HMI}}$ and $P_{\mathrm{fa}}$. Although an average allocation strategy has been used as a remedy, Ref. (20) noted that this fixed distribution mode may increase VPL. An averaged distribution strategy for risk allocation has two primary consequences: (i) Averaging risk can lead to under-allocation for high-risk hypotheses and over-allocation for low-risk hypotheses. Consequently, certain critical fault scenarios receive insufficient protection, which substantially raises the systemic risk of missed detections. (ii) When an averaged distribution strategy allocates unnecessary risk budgets to low-impact or unlikely fault hypotheses, it causes the VPL calculation to be inflated. This inflation occurs because these “redundant hypotheses” pull the VPL upward, making it a less precise and tighter bound than it could be. Various methods have been applied to distribute integrity risk and continuity risk reasonably (21, 22), wherein Ref. (21) suggested that optimizing the distribution of integrity risk can impact VPL. The ARAIM algorithm typically employs the weighted least squares (WLS) model for computing both full set and subset solutions, conducting integrity calculations based on the corresponding projection coefficients (23). However, as pointed out in Ref. (24), ARAIM may not fulfill the minimum VPL for integrity using the WLS model alone. To achieve a lower VPL, Ref. (25) suggested optimizing the full set solution, albeit at the cost of reduced positioning accuracy. The coefficient allocation was further analyzed on the integrity risk domain in Ref. (26), while a specified PL calculation was not provided. These studies primarily focused on a specific fault model and addressed only the optimization of the full set solution, offering limited effectiveness in optimizing VPL.

Based on the analysis, several key issues with current ARAIM methods can be identified. With an increasing number of GNSS satellites, the fault hypothesis space for multihypothesis ARAIM grows exponentially, which reduces the efficiency of integrity monitoring calculations and adversely affects real-time performance. Moreover, many ARAIM optimization schemes reduce the VPL by sacrificing positioning accuracy, which lowers system tolerance and leads to a higher false alert rate.

To address these challenges, this study proposes the optimal-ARAIM framework, which includes: (i) Maximum monitoring order mechanism: This component tackles the combinatorial explosion caused by multiple satellite faults. It prunes monitorable fault dimensions, thereby reducing computational complexity and improving real-time performance while maintaining integrity coverage. (ii) Minimax optimization-based estimator with risk allocation and specified accuracy: The proposed framework adopts a minimax optimization-based risk-allocation method to balance the risk assigned to each fault hypothesis. The VPL is subsequently optimized by refining the complete solution set, subject to a prescribed accuracy constraint, which minimizes VPL by adjusting the full-set solution without sacrificing accuracy. (iii) The optimal solution search in minmax optimization: The framework also addresses the optimal solution search problem in minmax optimization. Its core idea is to transform the inner maximization into a computable function *F*, in which the VPL under the worst-case fault mode serves as the output. This conversion reduces the original problem to minimizing *F*.

## Methodology

### Maximum monitoring mechanism

**Fig. 1.**
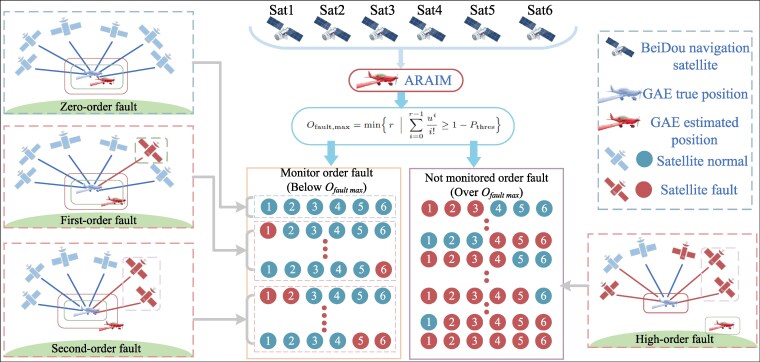
Maximum monitoring mechanism: the figure depicts the ARAIM algorithm fault grouping in GAE localization with the receiver capturing six satellites as an example. Firstly, the maximum monitoring fault order function is employed to calculate the $O_{\mathrm{fault},\max}=2$. Consequently, it is requisite to monitor the zero-order faults, the first-order faults, and the second-order faults. The left side of the figure indicates that it is essential to monitor these groupings, which are considered potential fault scenarios in the localization procedure. Fault groups exceeding $O_{\mathrm{fault},\max}$ need not be monitored, as depicted on the right side of the figure, encompassing 3rd, 4th, 5th, and 6th order faults.

The prerequisite for constructing MHSS-ARAIM is to ascertain the maximum number of fault hypotheses $N_{\mathrm{fault},\max}$. To determine a suitable $N_{\mathrm{fault},\max}$, one must first establish the maximum fault order, $O_{\mathrm{fault},\max}$. Knowing $O_{\mathrm{fault},\max}$ allows ARAIM to preset its groupings more effectively. For clarity, we conduct a GAV scenario utilizing the predefined number of satellites and $O_{\mathrm{fault},\max}$, as depicted in Fig. 1.

For reducing the number of hypotheses, we need to calculate $O_{\mathrm{fault},\max}$, based on the number of simultaneous faults. Hypothesis types are classified into fault groups as follows:

- Zero-order fault group (r=0): No fault occurs, and all visible satellites work normally.
- First-order fault group (r=1): Include single satellite fault and single constellation fault.
- Second-order fault group (r=2): Include dual-satellite faults, single-satellite and single-constellation faults, and dual-constellation faults.

Higher-order fault groups, like third-order and fourth-order, adhere to this pattern analogously. We initially enumerate the occurrence probabilities of zero-order, first-order, and second-order fault groups as follows:


(1) $$\left\{ \begin{array}{c} P_{0}=\prod_{k=1}^{N_{\mathrm{fault}}}(1-P_{\mathrm{fault},k}),\;P_{1}=P_{0}\sum_{k=1}^{N_{\mathrm{fault}}}\frac{P_{\mathrm{fault},k}}{1-P_{\mathrm{fault},k}}\;\\ P_{2}=P_{0}\sum_{k_{1}<k_{2}}^{N_{\mathrm{fault}}}\frac{P_{\mathrm{fault}k_{1}}}{1-P_{\mathrm{fault},k_{1}}}\frac{P_{\mathrm{fault},k_{2}}}{1-P_{\mathrm{fault},k_{2}}},\; \end{array}\right.$$

where, $P_{\mathrm{fault},k}$ denotes the probability of failure of the *k*th satellite (constellation); and $N_{\mathrm{fault}}=N_{\mathrm{sat}}+N_{\mathrm{const}}$. The derivation of Eq. 1 can be found in Chapter 7, Part A in the SI.

From Section 7B of SI, let $u=\sum_{k=1}^{N_{\mathrm{fault}}}P_{\mathrm{fault},k}$, we formulate the maximum monitoring fault order function as (see SI Section 7 for detailed derivation)


(2) $$O_{\mathrm{fault},\max}=\min\left\{r|\sum_{i=0}^{r-1}\frac{u^{i}}{i!}\geq 1-P_{\mathrm{thres}}\right\},$$

where $\varphi_{\mathrm{thres}}$ returns a positive integer starting from 0. To identify the appropriate value for $O_{\mathrm{fault},\max}$, it is essential that the aforementioned formula is satisfied.


(3) $$\left\{ \begin{array}{c} P_{\mathrm{HMI},M}=\sum_{s=1}^{O_{\mathrm{fault},\max}}P_{\mathrm{fault},s},P_{\mathrm{HMI},\mathrm{NM}}=P_{\mathrm{HMI}}-P_{\mathrm{HMI},M}\;\\ P_{\mathrm{HMI},\mathrm{new}}^{V}=P_{\mathrm{HMI}}^{V}\left(1-\frac{P_{\mathrm{HMI},\mathrm{NM}}}{P_{\mathrm{HMI}}^{V}}\right),\; \end{array}\right.$$

where, $P_{\mathrm{fault},s}$ denotes the occurrence probability of an *s*-order fault group; $P_{\mathrm{HMI},\mathrm{new}}^{V}$ indicates the reduction in overall $P_{\mathrm{HMI}}$ due to the existence of unmonitored hypothesis modes. Specific instructions can be found in Section 7C of the SI. Due to the maximum monitoring order mechanism, some potential low-probability faults are not included in the monitoring, rendering the original PHMI inaccurate. We propose dividing the PHMI into two parts: the total probability of monitored fault hypotheses and the PHMI of unmonitored fault hypotheses (calculated by subtracting the monitored portion from the total PHMI). The PHMI is then corrected proportionally based on these two parts.

### ARAIM integrated with solution separation

The fundamental observation models and positioning methods in satellite navigation are first reviewed. The pseudorange observation model relying on the BeiDou system and the solution derived through the utilization of the WLS are presented below:


(4) $$\left\{ \begin{array}{c} y=GX+\varepsilon ,\\ \hat{X}=(G{\;}^{T}WG{)}^{-1}G{\;}^{T}Wy, \end{array}\right.$$

wherein *G* is the geometric observation matrix with dimensions $N_{\mathrm{sat}}\times (3+N_{\mathrm{const}})$; *X* represents the estimated vector of dimensions $(3+N_{\mathrm{const}})\times 1$; $\hat{X}$ represents *X* obtained using WLS; ε is the noise component, $\varepsilon ∼N(0,W^{2})$; *W* is the diagonal weight matrix and its inverse is denoted as accuracy matrix $C=W{\;}^{-1}$ as presented in Section 2 of SI. The initial three components of *X* denote the East-North-Up position values, while the remaining components stand for the clock bias. The symbol $s=(G{\;}^{T}WG{)}^{-1}G{\;}^{T}W$ can be designated as the projection matrix of dimensions $(3+N_{\mathrm{const}})\times N_{\mathrm{sat}}$, representing the mapping coefficient from the observation domain to the state domain.

The WLS solution employing all observation data is termed the full set solution. When partial observation data are utilized for the WLS solution, it is designated as a subset solution (20). The subset concept is closely tied to the hypotheses mode and supposes there exist $N_{\mathrm{fault}}$ modes. Each mode encompasses an identity list (${\text{idx}}_{k},k=1,2\ldots ,N_{\mathrm{fault}}$), where ${\text{idx}}_{k}$ denotes fault-free information by the receiver being used. Let $s^{\left(\mathrm{full}\right)}$ be the full set of solution projection coefficients and $s^{\left(k\right)}$ be the subset of solution projection coefficients, we have


(5) $$\left\{ \begin{array}{c} \hat{X}{\;}_{3}^{\left(\mathrm{full}\right)}=s{\;}_{3}^{\left(\text{full}\right)}y=e{\;}_{3}^{T}(G{\;}^{T}W_{0}G{)}^{-1}G{\;}^{T}W_{0}y\\ \hat{X}{\;}_{3}^{\left(k\right)}=s{\;}_{3}^{\left(k\right)}y_{k}=e{\;}_{3}^{T}(G{\;}^{T}W_{k}G{)}^{-1}G{\;}^{T}W_{k}y_{k}, \end{array}\right.$$

where $e_{3}$ denotes the vertical extraction vector with $e_{3}=[0\;0\;1\;0{]}^{T}$ so that $\hat{X}{\;}_{3}=e{\;}_{3}^{T}\hat{X}$ holds for a single constellation; $y_{k}$ represents the subset input observation data; $W_{0}$ and $W_{k}$ represent the full set and subset weight diagonal matrices, respectively. For the subset *k*, $W_{k}$ is defined as follows:


(6) $$W_{k}(i,i)=\left\{ \begin{array}{cc} C{\;}^{-1}(k,k) & \text{if}\;k\;\text{is in}\;{\text{idx}}_{k}\\ 0 & \text{otherwise}, \end{array}\right.$$

where $W_{k}=E_{k}W_{0}$ with $E_{k}$ being a quasi-identity matrix that sets the positions of non-${\text{idx}}_{k}$ elements to zero. Then, we are able to construct the solution separation ${\text{SS}}_{k}$ and detection thresholds $T_{k,3}$ for subset *k*, as follows:


(7) $$\left\{ \begin{array}{c} {\text{SS}}_{k}=|\hat{X}{\;}_{3}^{\left(k\right)}-\hat{X}{\;}_{3}^{\left(\mathrm{full}\right)}|\\ T_{k,3}=K_{\mathrm{fa},k}\sigma_{\mathrm{ss},k}. \end{array}\right.$$

If $|\hat{X}{\;}_{3}^{\left(k\right)}-\hat{X}{\;}_{3}^{\left(\mathrm{full}\right)}|>T_{k,3}$, which indicates that the magnitude of solution separation exceeds the detection threshold, then the system issues a fault warning. When $|\hat{X}{\;}_{3}^{\left(k\right)}-\hat{X}{\;}_{3}^{\left(\mathrm{full}\right)}|\leq T_{k,3}$, the VPL is calculated as


(8) $$\left\{ \begin{array}{c} {\text{VPL}}_{k}=T_{k,3}+K_{\mathrm{md},k}\sigma_{k}+b_{k}\\ \text{VPL}=\max_{k}\;{\text{VPL}}_{k}, \end{array}\right.$$

where ${\text{VPL}}_{k}$ is calculated using the *k*th subset. Then, the VPL is determined by the maximum ${\text{VPL}}_{k}$ of all subsets. The parameters in (7) and (8) are explained as follows: $\sigma_{\mathrm{ss},k}$ represents standard deviation of ${\text{SS}}_{k}$; $K_{\mathrm{fa},k}$ is the continuity allocation factor for fault hypothesis *k*, $K_{\mathrm{fa}}=[K_{\mathrm{fa},1},K_{\mathrm{fa},2},\ldots ,K_{\mathrm{fa},N_{\max}}]$; $\sigma_{k}$ denotes the standard deviation of $\hat{X}{\;}_{3}^{\left(k\right)}$ and $b_{k}$ is the nominal error; $K_{\mathrm{md},k}$ is the integrity allocation factor with $K_{\mathrm{md}}=[K_{\mathrm{md},1},K_{\mathrm{md},2},\ldots ,K_{\mathrm{md},N_{\max}}]$. The above parameters are computed in the following ways:


(9) $$\left\{ \begin{array}{c} \sigma_{\mathrm{ss},k}=\sqrt{(s{\;}_{3}^{\left(\text{full}\right)}-s{\;}_{3}^{\left(k\right)})C(s{\;}_{3}^{\left(\text{full}\right)}-s{\;}_{3}^{\left(k\right)}{)}^{T}}\\ \sum_{k=0}^{N_{\mathrm{fault}}}2Q(K_{\mathrm{fa},k})=P_{\mathrm{fa},V},\sigma_{k}=\sqrt{s{\;}_{3}^{\left(k\right)}Cs{\;}_{3}^{\left(k\right)T}}\\ b_{k}=|s_{3}^{\left(k\right)}|b_{\mathrm{nom}},\sum_{k=0}^{N_{\mathrm{fault}}}P_{\mathrm{event},k}Q(K_{\mathrm{md},k})=P_{\mathrm{HMI}}^{V}, \end{array}\right.$$

where $b_{\mathrm{nom}}$ is derived from ISM; $P_{\mathrm{event},k}$ represents the prior probability of subset *k*; and *Q* is inversely normally distributed.

### VPL-oriented minimax estimator based on optimal-ARAIM

**Fig. 2.**
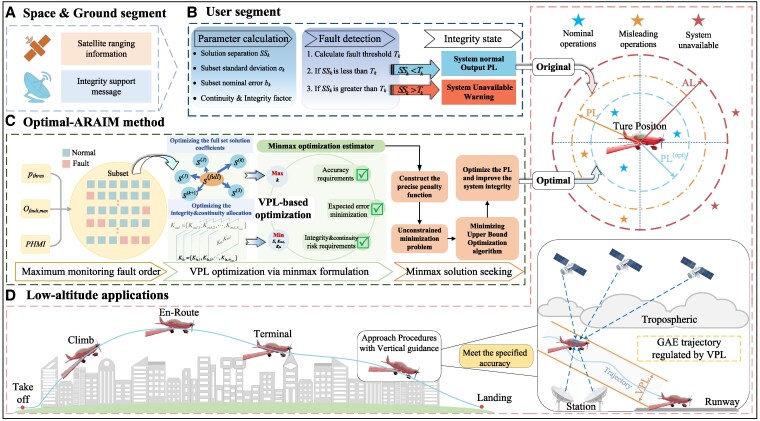
Overall framework of the Optimal-ARAIM method: A) ARAIM is segmented into a three-layer structure. The initial two layers pertain to the space segment and the ground segment. The space segment is characterized as satellite ranging data. The ground segment comprises the a priori fault information. B) The third-layer structure of ARAIM constitutes the user segment, which is partially dedicated to parameter computation and partially to fault detection and original PL calculation, but the PL values obtained are relatively loose. C) The Optimal-ARAIM algorithm has the full set solution acquired by WLS and the subset solution as its algorithm inputs. Subsequently, an optimization estimator for VPL is constructed. And the estimator is further transformed into solving the minmax estimator. Ultimately, the MUBO algorithm is employed to obtain the optimal solution of this estimator, thereby the PL values are more compact. D) In the application part, the GAE low-altitude flight scenario is presented. One of the problems that needs to be addressed in the LAE is how GAE can effectively avoid buildings during flight.

The overall flowchart of the Optimal-ARAIM method is depicted in Fig. 2. The goal of employing the WLS is to optimize the accuracy by minimizing the variance $\sigma {\;}_{\mathrm{best},V}^{2}=e{\;}_{3}^{T}(G{\;}_{0}^{T}W_{k}G_{0}{)}^{-1}e{\;}_{3}$. However, the WLS solution does not correspond to the optimal integrity. To obtain the optimal VPL for integrity, we require expanding (8) for the analysis using:


(10) $$\text{VPL}=\max_{k}K_{\mathrm{fa},k}\sigma_{\mathrm{ss},k}+K_{\mathrm{md},k}\sigma_{k}+b_{k}.$$

For a certain subset *k*, $b_{k}$ and $\sigma_{k}$ are fixed quantities by the third term of (9). From (7), it is evident that $s{\;}_{3}^{\left(k\right)}$ is determined by the subset weight matrix and hence remains unchangeable. Consequently, based on the foregoing analysis, three elements affect the magnitude of the VPL: (i) the continuity allocation factor $K_{\mathrm{fa},k}$ of each subset; (ii) the integrity allocation factor $K_{\mathrm{md},k}$ of each subset; and (iii) $\sigma_{\mathrm{ss},k}$, which is the standard deviation of ${\text{SS}}_{k}$ between $s{\;}_{3}^{\left(k\right)}$ and $s{\;}_{3}^{\left(\mathrm{full}\right)}$.

As stated in the introduction, we optimize both $K_{\mathrm{md}}$ and $k_{\mathrm{fa}}$. Meantime, to reducing $T_{k,3}$, it is necessary to decrease $\sigma_{\mathrm{ss},k}$. Based on the analysis of (10), $s{\;}_{3}^{\left(k\right)}$ is deterministic. Regarding $s{\;}_{3}^{\left(\mathrm{full}\right)}$, it is evident from the experiments in Section 3 of SI that employing $s{\;}_{3}^{\left(\mathrm{full}\right)}$ does not invariably lead to a minimum VPL. Suppose that $s{\;}_{3}^{\left(\text{opt}\right)}$, which is distinct from $s{\;}_{3}^{\left(\mathrm{full}\right)}$, is utilized to compute $\sigma_{\mathrm{ss},k}$, thereby enabling a smaller VPL and enabling $s{\;}_{3}^{\left(\mathrm{opt}\right)}$ to meet the LPV-200 and CAT-I accuracy criterion. This enables us to formulate a constrained estimation problem (24) that determines $s{\;}_{3}^{\left(\text{opt}\right)}$, $K_{\mathrm{md}}$ and $K_{\mathrm{fa}}$ through outer-loop minimization and subset *k* through inner-loop maximization:


(11) $$\min_{s{\;}_{3}^{\left(opt\right)},K_{\mathrm{md}},K_{\mathrm{fa}}}\max_{k}K_{\mathrm{fa},k}\sigma {\;}_{\mathrm{ss},k}^{\left(opt\right)}+K_{\mathrm{md},k}\sigma_{k}+b_{k}$$

$$\begin{array}{rl} \text{s.t.} & \sum_{k}^{N_{\max}}P_{\mathrm{fault},k}Q(K_{\mathrm{md},k})=P_{\mathrm{HMI},\mathrm{new}}^{V},\\ & \;2\sum_{k=0}^{N_{\mathrm{fault}}}Q(K_{\mathrm{fa},k})=P_{\mathrm{fa},V},\sigma {\;}_{\mathrm{opt},V}^{2}\leq \sigma {\;}_{\mathrm{req},V}^{2}, \end{array}$$

wherein $\sigma {\;}_{\mathrm{ss},k}^{\left(opt\right)}=\sqrt{(s{\;}_{3}^{\left(opt\right)}-s{\;}_{3}^{\left(k\right)})C(s{\;}_{3}^{\left(opt\right)}-s{\;}_{3}^{\left(k\right)}{)}^{T}}$. The first and second constraint are integrity and continuity risk requirements; the third is the accuracy constraint with $\sigma {\;}_{\mathrm{req},V}^{2}$ denoting the accuracy requirement, specified as 1.86 m in the vertical direction; $\sigma {\;}_{\mathrm{opt},V}^{2}$ represents the standard deviation of $s{\;}_{3}^{\left(opt\right)}$, given by $\sigma {\;}_{\mathrm{opt},V}^{2}=s{\;}_{3}^{\left(opt\right)}Cs{\;}_{3}^{(opt)T}$. Considering that the new estimator should minimize the expected error, one follows from model (4) to have


(12) $$E(s{\;}_{3}^{\left(opt\right)}y)-e{\;}_{3}^{T}x=(s{\;}_{3}^{\left(opt\right)}G-e{\;}_{3}^{T})x+E(s{\;}_{3}^{\left(opt\right)}\varepsilon ),$$

wherein the expectation of ε should be zero. Therefore, the following condition must be satisfied (24)


(13) $$s{\;}_{3}^{\left(opt\right)}G=e{\;}_{3}^{T}.$$

Only the minmax problem that satisfies the requirements of (13) can make the position error controllable. Consequently, the problem presented in (11) should incorporate (13) as a new constraint to guarantee its validity, as follows:


(14) $$\begin{array}{rl} & \min_{s{\;}_{3}^{\left(opt\right)},K_{\mathrm{md}},K_{\mathrm{fa}}}\max_{k}\;K_{\mathrm{fa},k}\sigma {\;}_{\mathrm{ss},k}^{\left(opt\right)}+K_{\mathrm{md},k}\sigma_{k}+b_{k}\\ \text{s.t.} & \sum_{k}^{N_{\max}}P_{\mathrm{fault},k}Q(K_{\mathrm{md},k})=P_{\mathrm{HMI},\mathrm{new}}^{V},\\ & 2\sum_{k}^{N_{\max}}Q(K_{\mathrm{fa},k})=P_{\mathrm{fa}}^{V},\;\sigma {\;}_{\mathrm{opt},V}^{2}\leq \sigma {\;}_{\mathrm{req},V}^{2},\;s{\;}_{3}^{\left(opt\right)}G=e{\;}_{3}^{T}. \end{array}$$

We refer to (14) as a minmax optimization estimator, employing the null-space transformation in Section 4 of SI as follows:


(15) $$\begin{array}{rl} \min_{x,y,t} & \;\max_{k}\;t_{k}\|x-a_{k}{\|}_{2}+y_{k}\\ \text{s.t.} & \sum_{k}^{N_{\max}}P_{\mathrm{fault},k}Q(\frac{y_{k}-b_{k}}{\sigma_{k}})=P_{\mathrm{HMI},\mathrm{new}}^{V},\\ & 2\sum_{k}^{N_{\max}}Q(t_{k})=P_{\mathrm{fa}}^{V},\;x^{T}x-(\sigma {\;}_{\mathrm{req},V}^{2}-\sigma {\;}_{\mathrm{best},V}^{2})\leq 0, \end{array}$$

where $x\in R^{N_{\mathrm{sat}}-N_{\mathrm{const}}-3}$ is $s{\;}_{3}^{\left(opt\right)}$ after a null-space transformation to get the decision variable form; and $y=[y_{1},y_{2},\ldots ,y_{N_{\max}}{]}^{T}$ is the simplified decision variable form of $K_{\mathrm{md}}$, $t=[t_{1},t_{2},\ldots ,t_{N_{\max}}{]}^{T}$ represents the simplified decision variable of $K_{\mathrm{fa}}$.

### Minmax solution seeking

We define a new function F(x,y) containing max based on the initial objective function and merge the new objective function into the following constraint condition


(16) $$\begin{array}{rl} \min_{x,y,t} & \;F(x,y,t)=\max_{k}f_{k}(x,y,t)\\ \text{s.t.} & \;f_{k}(x,y,t)=t_{k}\|x-a_{k}{\|}_{2}+y_{k},g(x)\leq 0,h(y,t)=0, \end{array}$$

where g(x) and h(y,t) are the inequality constraint and equality constraint in (15), respectively. To solve the above minmax estimator, an upper bound variable *u* (27) is introduced, thus transforming it into a minimization optimization problem, yielding


(17) $$\begin{array}{rl} \min_{x,y,t,u} & \;u\\ \text{s.t.} & \;F(x,y,t)\leq u,\;g(x)\leq 0,\;h(y,t)=0. \end{array}$$

This renders the solution of (17) equivalent to that of (16). By introducing the penalty function (28), the minmax problem can be rewritten as


(18) $$\begin{array}{rl} E(x,y,t,u;M,\rho ) & =\frac{1}{2}[(u-M{)}^{+}{]}^{2}\\ & \;+\frac{\rho }{2}\{\sum_{k}[(f_{k}(x,y,t)-u{)}^{+}{]}^{2}+[g^{+}(x){]}^{2}+h^{2}(y,t)\}, \end{array}$$

where $\Gamma (\mu {)}^{+}=\max\{\Gamma (\mu ),0\}$; M∈R is referred to as the approximation parameter; and ρ>0 is the constraint penalty factor. The first term represents the approximation component, and the second one constitutes the penalty component. By giving a definite value for *M* and *ρ*, (18) can be reformulated as an unconstrained minimization problem:


(19) $$\min_{x,y,u}E(x,y,t,u;M,\rho ).$$

With the theorems in Section 5 of SI, the solution in (19) converges to its optimum $\left(x^{*},y^{*},t^{*},u^{*}\right)$ in (17) by adjusting the value of *M*. Consequently, minimizing the upper bound *u* of F(x,y,t) can be handled by solving the minimization (19). The MUBO algorithm, which can efficiently obtain the optimal solution $\left(x^{*},y^{*},t^{*},u^{*}\right)$, is given as shown in Algorithm 1. The MUBO algorithm undertakes the dynamic update of the upper and lower bounds of the M-interval, thereby promoting the convergence of $\left(x_{i},y_{i},t_{i},u_{i}\right)$ to approach the optimal solution. Furthermore, through the verification of the value of *E* and the determination of whether $\left(x_{i},y_{i},t_{i},u_{i}\right)$ resides within the feasible domain of (17), it is guaranteed that the solution of (19) can meet the constraints of (17). To sum up, the MUBO algorithm achieves the optimal solution of the minmax optimization estimator (15). In the actual optimization, $K_{\mathrm{fa}}$, $K_{\mathrm{md}}$, and $s^{\left(\mathrm{full}\right)}$ use the MUBO algorithm with the idea of alternate optimization to ensure the stability of the solution and the convergence of the optimal VPL.

**Algorithm 1:** Minimum Upper Bound Optimization

**Require** ρ>0, $i_{\max,\text{iter}}$, $\left(x_{0},y_{0},t_{0}\right)$
**Ensure** Optimal solution (x,y,u) for Eq. 17
1: Initialize i←1; define $\beta_{0}=u_{0}=F(x_{0},y_{0},t_{0})$; define $\alpha_{0}<\min F(x,y,t)$; set $M_{0}\leftarrow (\alpha_{i}+\beta_{i})/2$
2: **While** True **do**
3: Solve $E(x_{i-1},\;y_{i-1},t_{i-1},\;u_{i-1};\;M_{i-1},\;\rho )$ in (19)
4: Increment i←i+1
5: **if** $i>i_{\max,\text{iter}}$ **then**
6: **return** $\left(x_{i},\;y_{i},\;t_{i},\;u_{i}\right)$ as the optimal solution for (17)
7: **end if**
8: Update $\left(x_{i},y_{i},t_{i},u_{i}\right)$ as the global solution to the current problem (19)
9: **if** $E(x_{i},\;y_{i},\;t_{i},\;u_{i};\;M_{i},\;\rho )=0$
10: Set $\alpha_{i}\leftarrow \alpha_{i-1}$, $\beta_{i}\leftarrow u_{i-1}$, $M_{i}\leftarrow (\alpha_{i}+\beta_{i})/2$
11: **else**
12: **if** $\left(x_{i},y_{i},u_{i}\right)$ is not a feasible point for (17)
13: Set $\alpha_{i}\leftarrow \max\{u_{i},\;M_{i}\}$, $\beta_{i}\leftarrow \beta_{i-1}$, $M_{i}\leftarrow (\alpha_{i}+\beta_{i})/2$
14: **else**
15: **return** $\left(x_{i},\;y_{i},\;t_{i},\;u_{i}\right)$ as the optimal solution for (17)
16: **end if**
17: **end if**
18: **end while**

## Results

### Experimental setup

The assessment is categorized into three parts: evaluations on the maximum monitoring mechanism, BeiDou verification and DFMC (BeiDou + GPS) verification. To verify the performance of Optimal ARAIM, we compared it with Baseline-ARAIM (20), which represents the classic MHSS-ARAIM, and Simple-ARAIM (25), which exclusively optimizes the coefficients of the full set of solutions. To evaluate the effectiveness of IM methods, a key indicator for evaluating system integrity is introduced as Ref. (29) $\text{EMT}=\max T_{k,3}\;|\;P_{\mathrm{fault},k}>10^{-5}$, which must be <15 m for LPV-200 and CAT-I. EMT sets an upper limit on the fault threshold for each fault hypothesis. If the indicator exceeds 15 m, the system is deemed unavailable.

**Fig. 3.**
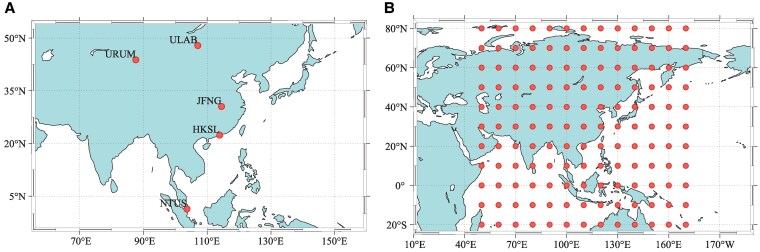
Observation station coordinates and regional test point locations: A) Observation data from day of year (DOY) 49 to 51 in 2024 were utilized, with a sampling interval of 30 s, resulting in a total of 8,640 epochs. B) A total of 143 test coordinate points were simulated. Almanac data from 931 BeiDou weeks (2023 November 5) was selected to simulate the VPL distribution throughout a day, calculated at 5 min intervals with a total of 288 time points.

We evaluated the maximum monitoring mechanism: simulating single, double, and three constellation satellite configurations to verify the effectiveness of the grouping mechanism. For the observation data, five observation stations were selected evenly across Asia, as depicted in Fig. 3A: (i) Wuhan (JFNG), (ii) Urumqi (URUM), (iii) Hong Kong (HKSL), (iv) Mongolia (ULAB), and (v) Singapore (NTUS). These stations can receive the pseudorange signals from BeiDou-2 and BeiDou-3 satellites. For the prediction of almanac, the test area spans from $50{\;}^{\circ }$E to $170{\;}^{\circ }$ E longitude and from $20{\;}^{\circ }$S to $80{\;}^{\circ }$N latitude, covering most of Asia. Furthermore, we subdivide the test area into $10{\;}^{\circ }\times 10{\;}^{\circ }$ grids, as shown in Fig. 3B. Notably, the observation data are sourced from the public observation data of the International GNSS Service stations (30) and the BeiDou almanac data are available from the China Satellite Navigation Office (31).

In verifying the GNSS (BeiDou+GPS) dual system, we utilize almanac data of BeiDou-3 and GPS to assess the system availability, which can comprehensively evaluate the VPL, EMT, and accuracy of the system. By quantifying the availability of navigation solutions, coverage indicators that satisfy CAT-I can be obtained as


(20) $$\left\{ \begin{array}{c} \text{Csp}(j)=\frac{1}{n}\sum_{i=1}^{n}[\text{VPL}(i)\leq 15\cap \text{EMT}(i)\leq 15\cap \sigma_{\mathrm{req}}(i)\leq 1.86]\;\\ \text{Gsp}=\frac{1}{m}\sum_{\;j=1}^{m}[\text{Csp}(j)\times 100\%\geq 99.5\%],\; \end{array}\right.$$

where Csp denotes single-point availability coverage over the time series *i*; Gsp represents global availability coverage across the coordinate series *j*. The test area extends from $-170{\;}^{\circ }$ to $180{\;}^{\circ }$ longitude and $-80{\;}^{\circ }$ to $80{\;}^{\circ }$ latitude, covering most of the globe. This area is segmented into a grid with dimensions of $10{\;}^{\circ }\times 10{\;}^{\circ }$, amounting to 612 coordinate points, which is comparable to those illustrated in Fig. 3B yet on an enlarged scale. Almanac data from 917 BeiDou week and 2,273 GPS week (2023 August 1) were selected, with calculations carried out every 5 min to simulate daily availability, VPL, EMT, and accuracy distributions across 288 time points.

### Evaluations on maximum monitoring mechanism

To validate the function of $O_{\mathrm{fault},\max}$ and the impact of $P_{\mathrm{HMI},\mathrm{NM}}$ on the integrity risk, we have conducted a simple case experiment involving three constellation combinations. The parameter settings are the same as those in Table S1 in Section 1 of SI, and the experimental outcomes are presented in Table 1.

**Table 1.** Comparison regarding the count of monitored orders and the modifications of $P_{\mathrm{HMI}}$.

Constellations	Number of satellites	Maximum monitoring fault order function			Total fault hypotheses		$P_{HMI}$		$P_{HMI}^{V}$	
		$P_{thres}$	$O_{fault,max}$	Number of hypotheses	Number of hypotheses	hypothesis reduction ratio	$P_{HMI,NM}$	Rate (%)	$P_{HMI,new}^{V}$	Rate (%)
BeiDou-3	8	$9\times 10^{-8}$	1	10	130	64.61	$1.28\times 10^{-8}$	12.80	$7.85\times 10^{-8}$	87.20
BeiDou-2	8	$1\times 10^{-8}$	2	211	1,351	84.38	$9.90\times 10^{-9}$	9.90	$8.11\times 10^{-8}$	90.10
BeiDou-3	10									
BeiDou-2	7	$9\times 10^{-9}$	2	562	6,018	90.66	$8.91\times 10^{-9}$	8.91%	$8.20\times 10^{-8}$	91.10
BeiDou-3	10									
GPS	13									

In Table 1, $P_{\mathrm{thres}}$ in column 3 is determined based on the data in columns 1 and 2, which represent the number of constellations and satellites, respectively. $O_{\mathrm{fault},\max}$ in column 4 is computed by (2), and column 5 contains the number of hypotheses calculated by the value of $O_{\mathrm{fault},\max}$. Column 6 represents the number of hypotheses obtained by fixing $O_{\mathrm{fault},\max}=3$, which is a common practice in traditional RAIM. Column 7 displays the percentage reduction calculated between columns 5 and 6. The number of hypotheses computed using $\varphi_{\mathrm{thres}}$ is decreased compared to that computed in the fixed method of RAIM. The data in column 8 are computed based on the second term of (3) and indicates the probability of occurrence of the unmonitored failure mode. Column 9 presents the percentage of occupied $P_{\mathrm{HMI}}$. It is observable that the percentage of occupied is relatively low. Columns 10 and 11 serve to validate the third term of (3). Column 10 contains the corrected $P_{\mathrm{HMI},\mathrm{new}}^{V}$, and column 11 shows the ratio of the corrected $P_{\mathrm{HMI}}$ to the original $P_{\mathrm{HMI}}$. Overall, the calculation of $O_{\mathrm{fault},\max}$ through the utilization of $\varphi_{\mathrm{thres}}$ is achievable to reduce the number of hypotheses for ARAIM in the DFMC scenario.

### Evaluations on BeiDou

#### Observational data results

**Fig. 4.**
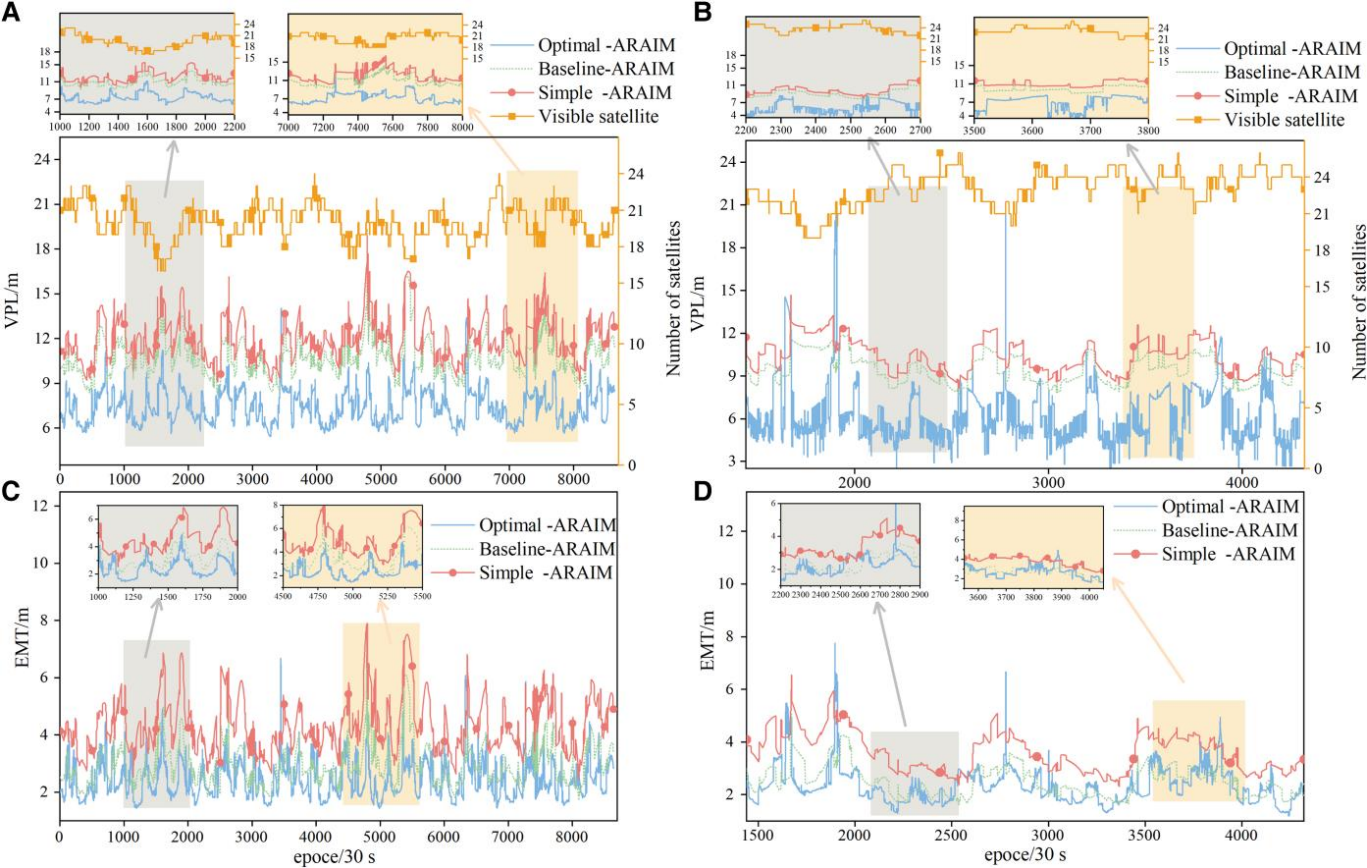
The Epoch-by-epoch VPL and EMT results of the observation station: A and B) depict the VPL estimation outcomes for two stations, JFNG and ULAB. To more effectively illustrate the performance of the algorithm, JFNG demonstrates the test data for all epochs within 3 days, whereas ULAB showcases the test data for 1 day. C and D) display the EMT estimation results for both JFNG and ULAB stations in a manner similar to that of (A) and (B).

For the sake of clarity, only two stations, JFNG and ULAB, are chosen to obtain VPL and EMT, while findings from these two stations apply to those from the others. The test results of VPL and EMT from the two stations are shown in Fig. 4. Upon analysis of results, we find that the algorithm of Optimal-ARAIM demonstrates better performance, with notable advantages in integrity monitoring. Regarding the VPL, baseline-ARAIM performs slightly better than Simple-ARAIM. But the Optimal-ARAIM obtains VPL values that are 2 to 3 m lower, ensuring that the vertical position result remains within a smaller protection level. Besides, the EMT estimated values obtained by the Optimal-ARAIM are reduced by ∼0.5 to 1 m compared to others.

**Fig. 5.**
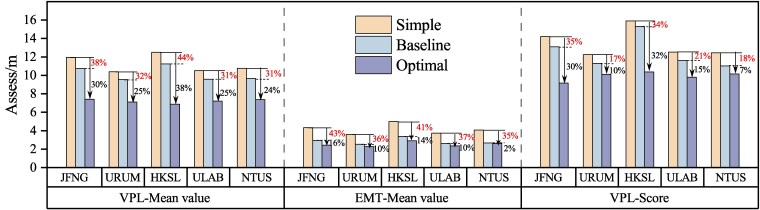
Mean results of DOY 49–51 at five stations: The histogram on the left depicts the statistics of VPL and the histogram on the right shows the statistics of EMT. The comparative results between Optimal-ARAIM and (Baseline/Simple)-ARAIM and algorithms are labeled in red and black, respectively. To evaluate the statistical performance of the algorithm, we additionally introduced the VPL score for comparison, calculated as the average of the 50th, 95th, and 99th percentiles of VPL.

Figure 5 presents the mean results of VPL and EMT for each station, as well as the VPL score at each station. In the statistical results of VPL and EMT presented in Fig. 5, the Optimal-ARAIM demonstrates an improvement of >40% over the Simple-ARAIM and more than 30% over the Baselinel-ARAIM, indicating its superior VPL estimation performance. Additionally, in terms of statistical significance, all three percentiles (50th, 95th, and 99th) of VPL outperform the baseline algorithm, demonstrating the stability of the VPL distribution calculated by Optimal-ARAIM, with few statistically significant false positives or missed detections.

#### Almanac data results

**Fig. 6.**
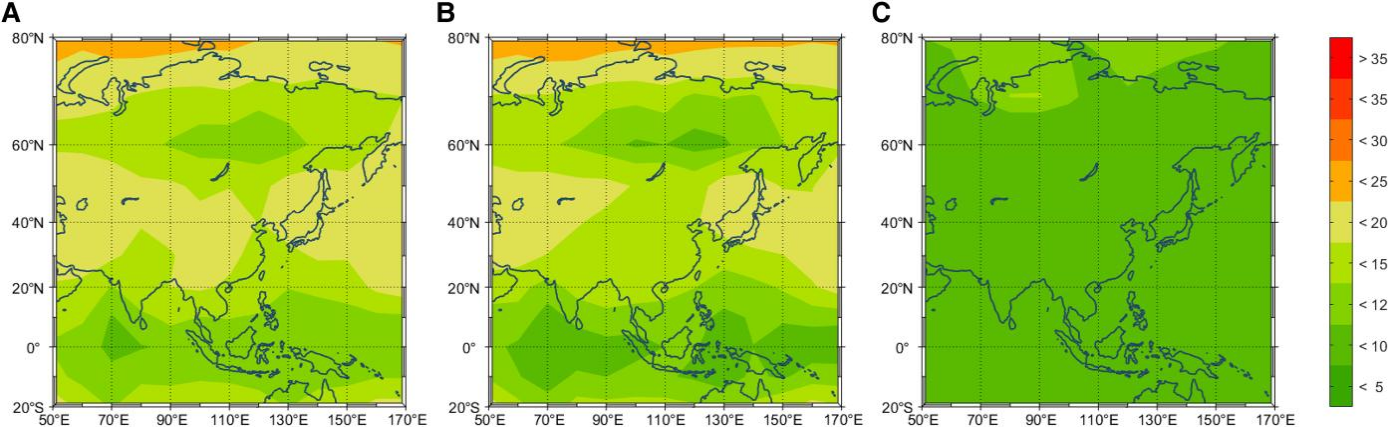
VPL distribution prediction in the Asian region: A) VPL <12 m is concentrated in the region from $20{\;}^{\circ }$S to $20{\;}^{\circ }$N, while most of the region from $20{\;}^{\circ }$N to $80{\;}^{\circ }$N is 15–20 m. B) VPL <10 m is only in the region from $20{\;}^{\circ }$S to $20{\;}^{\circ }$N, and most of the region from $20{\;}^{\circ }$N to $80{\;}^{\circ }$N is 10–15 m. C) VPL <10 m covers the majority of the test area, with only localized geographic areas having VPL between 10 and 20 m.

Using almanac data, we obtain the VPL of the Asian region predicted by three algorithms as shown in Fig. 6. It is seen that the VPL distribution of the Optimal-ARAIM achieves favorable results across most areas of Asia, wherein VPL estimation values are below 10 m in the range of $20{\;}^{\circ }$S to $80{\;}^{\circ }$N. In contrast, the Baseline-ARAIM only maintains VPL values <10 m in the equatorial region, and the Simple-ARAIM has an even smaller coverage area below the same threshold. These simulation results highlight the advantages of the proposed Optimal-ARAIM and demonstrate the reliability of using almanac data for VPL estimation.

#### Accuracy and parameter sensitivity analysis

**Fig. 7.**
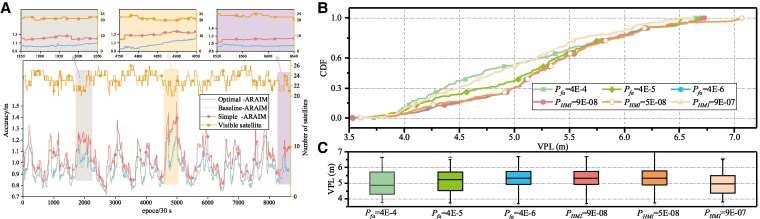
Positioning accuracy analysis under ARAIM algorithm: A) The horizontal axis represents the epoch (30 s), and the left vertical axis represents the Accuracy (m). In addition, the right vertical axis represents the number of available satellites. The accuracy profile of Optimal-ARAIM is slightly larger than that of Baseline-ARAIM for most calendar elements, but it is not obvious in the figure, so three zoomed-in subplots are given at epochs 1,850–2,050, 4,750–4,950, and 8,520–8,640 to aid in illustration. B) To validate the sensitivity of Optimal-ARAIM to statistical parameters, we set three different groups of parameters for $P_{\mathrm{fa}}$ and $P_{\mathrm{PHMI}}$ to calculate the corresponding VPL, with results presented in the form of cumulative distribution functions. C) We further plotted boxplots for the six experimental groups from (B).

The accuracy for station URUM localization under three algorithms is shown in Fig. 7A. Three algorithms all meet the CAI-I accuracy requirement, and the Baseline-ARAIM achieves the highest accuracy due to its use of the least squares estimator. Besides, the Optimal-ARAIM method is slightly less effective, while the Simple-ARAIM method has relatively poor accuracy. The VPL distributions calculated by Optimal-ARAIM using different statistical parameters ($P_{\mathrm{fa}}$ and $P_{\mathrm{PHMI}}$) are shown in Fig. 7B and C. It can be observed that Optimal-ARAIM achieves similar VPL distributions, indicating that the algorithm is moderately robust against variations in PLs caused by parameter changes. The same conclusion can be drawn from the mean and variance of the boxplots.

### Evaluations on GNSS under DFMC

**Fig. 8.**
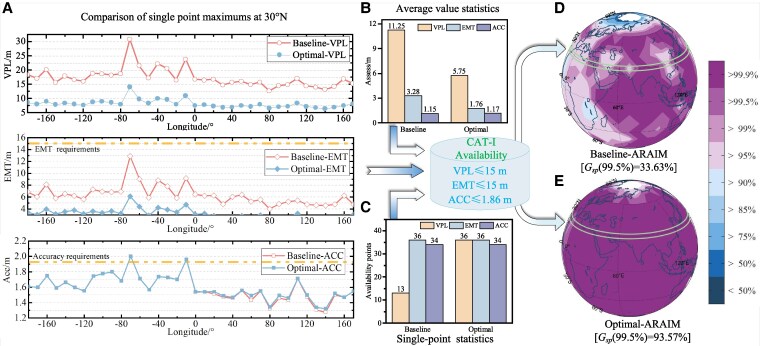
Results of global availability under Baseline-ARAIM and Optimal-ARAIM: A–C) involve the selection of simulated base station points at $30{\;}^{\circ }$N latitude for analysis, and D, E) presents the global availability outcomes. A total of 36 base station points are situated at $30{\;}^{\circ }$N, with each base station point having 288 simulated data points. The test results of other base stations are presented in Section 5 of the SI.

From the analysis in the previous section, it can be seen that the Simple-ARAIM algorithm exhibits a clear inferiority compared to the other two algorithms. Therefore, we concentrate only on comparing Baseline-ARAIM with the Optimal-ARAIM in this section. The almanac data results for the global region are presented in Fig. 8, and in conjunction with Figs. S1 and S2 of Section 6 of the SI. Below are the evaluation results for each set. The global availability outcomes are derived by employing the ARAIM algorithm for 612 simulated base stations. Baseline-ARAIM only achieved 99.5% coverage stands at 33.63%. But the Optimal-ARAIM obtained 99.5% coverage amounts to 93.57%.

1. The VPL, EMT, and accuracy results of the GNSS simulation conducted at $30{\;}^{\circ }$N are presented in Fig. 8A–C, respectively. The VPL and EMT test results obtained by the Optimal-ARAIM algorithm are better than those obtained by the Baseline-ARAIM algorithm in terms of maximum, average, and coverage. In particular, the number of stations with VPL ≤ 15 m estimated by Baseline-ARAIM is only 13, whereas by Optimal-ARAIM it is 36 in Fig. 8C. The low global coverage for Baseline-ARAIM largely results from the fact that the VPL value does not satisfy the CAI-I requirement.
2. The availability distributions in Fig. 8D and E (combined with Fig. S1A and B in Section 6 of SI) show that the Optimal-ARAIM algorithm improves availability by ∼40% compared to Baseline-ARAIM, especially in non-Asian regions. Using GPS+BeiDou-3 almanac data, the Optimal algorithm achieves excellent global availability performance. Regarding VPL distribution in Fig. S1C and D, Baseline-ARAIM maintains VPL estimated value below 15 m predominantly in Asia, whereas Optimal-ARAIM predicts VPL below 15 m across most test regions.
3. Regarding the EMT distribution depicted in Fig. S2A and B of SI, both algorithms fulfill the CAT-I requirement of having EMT ∼15 m. Typically, Optimal-ARAIM attains EMT values below 6 m, while Baseline-ARAIM remains below 9 m. The accuracy distribution illustrated in Fig. S2C and D of the SI also indicates that both algorithms comply with the accuracy requirement of 1.86 m. In local areas like $50{\;}^{\circ }$S to $60{\;}^{\circ }$S latitude, Optimal-ARAIM performs less effectively than Baseline-ARAIM. In conclusion, Optimal-ARAIM showcases advantages in estimating VPL. It effectively augments the availability of the navigation system.

## Discussion

Experimental results confirm the effectiveness of the proposed Optimal-ARAIM framework in addressing shortcomings of traditional BeiDou/GNSS integrity monitoring methods. Specifically, fixed-site calculations show that Optimal-ARAIM achieves lower overall and average VPL compared with previous studies (32, 33). Similarly, for the Asian region, our VPL values outperform those studied in Ref. (32, 34). Globally, the improvement extends, surpassing that presented in Ref. (24). Furthermore, from an availability standpoint, our study addresses a gap in previous research (23, 35) by providing a comprehensive evaluation of global availability.

These improvements are mainly due to two synergistic mechanisms implemented within Optimal-ARAIM. First, the maximum monitoring order mechanism effectively prunes the combinatorial explosion of fault hypotheses, improving real-time performance and computational efficiency for dynamic navigation applications. Second, the minmax optimization-based risk allocation strategy resolves the VPL conservatism seen in previous methods. By integrating risk measurement into the objective function and constructing penalty functions to ensure convexity and convergence, integrity and continuity risks are allocated intelligently without reducing positioning accuracy. Consequently, Optimal-ARAIM attains a tighter VPL while maintaining safety constraints, compared with many existing ARAIM optimization approaches.

## Conclusion

This study developed and validated the Optimal-ARAIM framework, addressing key challenges in current GNSS integrity monitoring. We effectively mitigated the combinatorial explosion of fault hypotheses by introducing a maximum monitoring mechanism. Integrity performance was further improved through a minmax optimization-based risk allocation strategy incorporating an accuracy requirement. The proposed framework successfully reduces the VPL without compromising positioning accuracy, representing a crucial improvement over existing methods. This enhanced integrity support capability is essential for navigation systems to meet the stringent certification requirements of safety-critical applications.

Although the experiment yielded promising results, performance evaluation remains insufficiently realistic, as it was conducted solely in controlled environments. Extensive real-world testing is crucial for improving the algorithm’s robustness and accuracy. Our future work will incorporate additional noise sources, including atmospheric delays and multipath. In addition, the algorithms need to be studied in terms of detection, identification, and adaptation.

## Supplementary Material

pgaf366_Supplementary_Data

## Data Availability

The datasets generated and analyzed during the current study are available in the Zenodo repository, 10.5281/zenodo.16397654.
