# Supplementary material for: Enhancing BeiDou/GNSS integrity with minmax optimization
Source: PNAS Nexus. 2025 Nov 14;4(11):pgaf366. doi: 10.1093/pnasnexus/pgaf366 (PMC12661408; doi:10.1093/pnasnexus/pgaf366)
Supplement: pgaf366_Supplementary_Data [file pgaf366_supplementary_data.pdf]

Supporting Information for

Enhancing BeiDou/GNSS Integrity with Minmax Optimization

Jingsong Qiu, Ci Chen, Siyu Lei, Yi Lyu, and Shengli Xie

E-mail: ci.chen@gdut.edu.cn, shlxie@gdut.edu.cn

This PDF file includes:

Supporting text
Figs. S1 to S3
Table S1
SI References

Supporting Information Text

1. Integrity parameter settings

The fault core parameters derived from long-term ground network observations must be obtained before using the ARAIM method. Such a method assesses whether the system is faulty and calculates the protection level according to the integrity parameter settings. Integrity parameter encompasses two categories: ISM and integrity constants, some of which are specified (1) in the Table S1.

Table S1. Integrity parameter settings

Category	Name	Description	Settings
Integrity Support Messages	$\sigma_{\text{URA},i}$	Standard deviation caused by clock and ephemeris of satellite i used to calculate accuracy and continuity	1
	$\sigma_{\text{URE},i}$	Standard deviation caused by clock and ephemeris of satellite i used to calculate integrity	$\frac{2}{3} \times \sigma_{\text{URA},i}$
	$b_{\text{nom},i}$	maximum nominal bias of satellite i	0.75
	$P_{\text{sat},i}$	Prior failure probability of satellite i	1×10^{-5}
	$P_{\text{const},j}$	Prior failure probability of constellation j	1×10^{-8}
Integrity constants	P_{HMI}	Probability of hazardously misleading information	1×10^{-7}
	P^V_{HMI}	Probability of PHMI assigned to the vertical direction	1×10^{-8}
	P_{thres}	Probability of integrity for not monitored failure modes	9×10^{-8}
	$P_{\text{fa},V}$	Continuity risks allocated to the vertical	3.9×10^{-6}

2. Accuracy matrix definition

The weighted least squares method needs to design the accuracy matrix to obtain the model solution. According to Eq. (1), accuracy matrix C is defined as $C = W^{-1}$ with each entry satisfying(1)

$$C(i,i) = \sigma^2_{\text{URE},i} + \sigma^2_{\text{tropo},i} + \sigma^2_{\text{user},i}, \tag{S-1}$$

where, $\sigma^2_{\text{URE},i}$ is provided by ISM; $\sigma^2_{\text{tropo},i}$ represents the tropospheric delay model; and $\sigma^2_{\text{user},i}$ accounts for the multipath and noise error experienced by the receiver for $i = 1, 2...., N_{\text{sat}}$, specified as

$$\begin{cases} \sigma_{\text{tropo},i} & = 0.12012/\sqrt{0.002001 + (\sin(\frac{\pi\theta}{180}))^2} \\ \sigma_{\text{user},i} & = \sqrt{(f^4_{B1} + f^4_{B3})/(f^2_{B1} - f^2_{B3})^2} * \sqrt{(\sigma_{\text{mp}})^2 + (\sigma_{\text{noise}})^2} \\ \sigma_{\text{mp}} & = 0.13 + 0.53e^{(-\theta/10^\circ)} \\ \sigma_{\text{noise}} & = 0.15 + 0.43e^{(-\theta/6.9^\circ)}, \end{cases} \tag{S-2}$$

where, f_{B1} and f_{B3} represent the pseudorange frequencies of BeiDou B1I and B3I; and θ is the satellite elevation angle.

3. VPL under different least squares solutions

To illustrate that the VPL derived from the WLS solution is typically not the minimal one, we employ actual observation data for demonstration. The observation data is sourced from the BeiDou pseudorange data gathered by the JFNG station on January 15, 2024. The geometric matrix is presented as follows:

$$G^T = \begin{bmatrix} -0.728 & 0.260 & -0.206 & -0.871 & 0.625 & 0.325 & 0.236 & 0.319 & -0.556 & 0.337 & 0.202 & -0.174 & -0.016 & 0.137 & 0.406 & -0.590 & -0.681 & -0.833 \\ -0.216 & -0.751 & -0.599 & -0.009 & -0.717 & -0.211 & 0.256 & -0.233 & 0.125 & -0.735 & -0.469 & -0.007 & 0.639 & -0.364 & -0.667 & -0.621 & -0.293 & 0.528 \\ -0.650 & -0.607 & -0.774 & -0.491 & -0.310 & -0.922 & -0.937 & -0.919 & -0.822 & -0.588 & -0.860 & -0.985 & -0.769 & -0.921 & -0.625 & -0.56 & -0.671 & -0.166 \\ 1 & 1 & 1 & 1 & 1 & 1 & 1 & 1 & 1 & 1 & 1 & 1 & 1 & 1 & 1 & 1 & 1 & 1 \end{bmatrix}.$$

The full set solution derived by the WLS model includes 18 available satellites. It is assumed that the observation data corresponding to the last row of the above matrix has a fault, while the first 17 satellites are fault-free. According to Eq. (9), the VPL can be seen to be no less than

$$VPL \geq K_{\text{fa,fault-free}}\sigma_{\text{ss,fault-free}} + K_{\text{md,fault-free}}\sigma_{\text{fault-free}} + b_{\text{fault-free}}, \tag{S-3}$$

where $\sigma_{\text{fault-free}}$ represents the variance of the fault-free part; while $K_{\text{md,fault-free}}$ and $K_{\text{fa,fault-free}}$ stand for the continuity and integrity distribution factors of the fault-free part, respectively. The average distribution approach is adopted, and there are 18 fault modes in total. Parameter Settings are consistent with Table S1, with the accuracy matrix C being the unit matrix, the value on the right-hand side of Eq. (S-3) is computed to be

$$\begin{aligned} & K_{\text{fa,fault-free}}\sigma_{\text{ss,fault-free}} + K_{\text{md,fault-free}}\sigma_{\text{fault-free}} + b_{\text{fault-free}} \\ & = 5.18 \times 0.69 + 2.36 \times 1.66 + 3.08 = 11.33. \end{aligned} \tag{S-4}$$

At this juncture, the VPL of the system is no less than 11.33 meters. However, if we consider using the fault-free part as the complete solution for VPL computation, that is, setting T_0 in the second formula of Eq. (8) to 0 (where the standard deviation of solution separation σ_{ss} is 0), the VPL can be calculated employing the first of Eq. (9) as follows:

$$\begin{aligned} VPL_{\text{fault-free}} &= T_0 + K_{\text{md},0}\sigma_{\text{fault-free}} + b_{\text{fault-free}} \\ &= K_{\text{fa},k}\sigma_{\text{ss},k} + Q^{-1}(\frac{P_{\text{HMI}}}{2})\sigma_{\text{fault-free}} + \sigma_{\text{fault-free}} \\ &= 0 + 2.36 \times 1.66 + 3.08 = 8.88. \end{aligned} \quad \text{[S-5]}$$

This simple example demonstrates that the VPL calculated using the most precise solution may not be optimal; the VPL can achieve a smaller value under non-optimal conditions.

4. Null space transformation and objective function transformation

A. Null space transformation. Building on the minimax optimization estimator Eq. (15), its form can be further simplified. The decision variable $s_3^{(\text{opt})}$ can be transformed using the null space transformation method(2) to satisfy constraint Eq. (14). Let the null space of G_0^T be U. For any feasible $s_3^{(\text{opt})}$, there exists a column vector z ($z \in R^{N_{\text{sat}}-N_{\text{const}}-3}$) can be expressed as

$$s_3^{(\text{opt})} = s_3^{(\text{full})} + (Uz)^T. \quad \text{[S-6]}$$

The method for finding the null space U is shown in Section 4.B. Substituting into Eq. (14), we have

$$e_3^T = (s_3^{(\text{full})} + (Uz)^T)G_0 = s_3^{(\text{full})}G_0 + z^T U^T G_0. \quad \text{[S-7]}$$

Since U is the null space of G_0^T, we have $G_0^T U = U^T G_0 = 0$. Thus, the second term of the Eq. (S-7) is zero. Combined with the first of Eq. (6), we obtain

$$e_3^T = s_3^{(\text{full})}G_0 = e_3^T(G_0^T W_0 G_0)^{-1}G_0^T W_0 G_0 = e_3^T. \quad \text{[S-8]}$$

For all vectors z, the third constraint of the minmax optimization estimator is satisfied. For the subset coefficient $s_3^{(k)}$, the difference vector with $s_3^{(\text{full})}$ naturally falls into the null space of G_0^T. Hence, there exists a column vector z_k ($z_k \in R^{N_{\text{sat}}-N_{\text{const}}-3}$) such that:

$$s_3^{(k)} - s_3^{(\text{full})} = (Uz_k)^T. \quad \text{[S-9]}$$

Note that z_k is a subset of the known transform vector. Further analysis of the objective function shows that the constant terms U, C can be simplified by using square root decomposition and transforming variables

$$\begin{cases} U^T C U = D^T D \\ a_k = Dz_k, x = Dz \\ y_k = K_{\text{md},k}\sigma_k + b_k, t_k = K_{\text{fa},k}, \end{cases} \quad \text{[S-10]}$$

where $y = [y_1, y_2, \cdots, y_{N_{\max}}]^T$. The details of variable transformation and constraint modifications from Eq. (S-6) to Eq. (S-11) are provided in Section 4.C. In summary, Eq. (15) transforms into

$$\min_{x,y,t} \quad \max_k t_k\|x - a_k\|_2 + y_k \quad \text{[S-11]}$$

$$\text{s.t.} \quad \sum_k^{N_{\max}} P_{\text{fault},k}Q(\frac{y_k - b_k}{\sigma_k}) - P_{\text{HMI}}^{V,\text{new}} = 0, \quad x^T x - K_{\text{fa}}^2(\sigma_{\text{req},V}^2 - \sigma_{\text{best},V}^2) \le 0.$$

A simplified form of the minimax optimization estimator is obtained through these transformations. To achieve the global optimal solution of this estimator, we outline the framework for obtaining global optimization in the next section.

B. Null space analysis. As described in Section 4.A, we require obtaining the null space U ($U \in R^{N_{\text{sat}} \times (N_{\text{sat}}-N_{\text{const}}-3)}$) of G^T such that $G^T U = 0$. This requires solving for the right null space U of G^T, which is equivalent to solving for the left null space U^T of the matrix G. From Eq. (1), G is of size $N_{\text{sat}} \times M$, where M represents the number of constellations plus 3. We obtain

$$G = L_G V_G R_G, \quad \text{[S-12]}$$

where L_G is an $N_{\text{sat}} \times N_{\text{sat}}$ matrix, R_G is an $M \times M$ matrix, and V_G is an $N_{\text{sat}} \times M$ matrix where the first M rows form a diagonal matrix and the remaining rows are all zeros. The L_G matrix is column-partitioned as

$$L_G = \left[L_{G,N_{\text{sat}}\times M} \quad L_{G,N_{\text{sat}}\times(N_{\text{sat}}-M)}\right], \quad \text{[S-13]}$$

in this expression, the column blocks represent the first M columns and the last $N_{\text{sat}} - M$ columns of L_G. Transposing $L_{G,N_{\text{sat}}\times M}$ to obtain $L_{G,N_{\text{sat}}\times M}^T$ and then multiplying by G yields

$$\begin{aligned}
&L_{G,N_{\text{sat}}\times M}^T G \\
=&L_{G,N_{\text{sat}}\times M}^T L_G V_G R_G \\
=&L_{G,N_{\text{sat}}\times M}^T \begin{bmatrix} L_{G,N_{\text{sat}}\times M} & L_{G,N_{\text{sat}}\times (N_{\text{sat}}-M)} \end{bmatrix} V_G R_G \\
=&L_{G,N_{\text{sat}}\times M}^T \begin{bmatrix} L_{G,N_{\text{sat}}\times M} & L_{G,N_{\text{sat}}\times (N_{\text{sat}}-M)} \end{bmatrix} \begin{bmatrix} V_{G,M\times M} \\ 0_{(N_{\text{sat}}-M)\times M} \end{bmatrix} V_G R_G \\
=&0_{(N_{\text{sat}}-M)\times M}.
\end{aligned} \tag{S-14}$$

From the Eq. (S-14), we can see that $L_{G,N_{\text{sat}}\times M}^T$ is the left null matrix of G, indicating that $L_{G,N_{\text{sat}}\times M}$ is the right null matrix of G^T. Due to the properties of singular value decomposition, the columns of the L_G matrix are mutually orthogonal, meaning the space formed by the columns of $L_{G,N_{\text{sat}}\times M}$ is the null space of G^T.

C. Objective function and constraint transformation. Substituting Eq. (S-10) into Eq. (15), we transform the objective function to

$$\begin{aligned}
&K_{\text{fa},k}\sqrt{(s_3^{(\text{full})} - s_3^{(k)} + (Uz)^T)C(s_3^{(\text{full})} - s_3^{(k)} + (Uz)^T)^T} + K_{\text{md},k}\sigma_k + b_k \\
=&K_{\text{fa},k}\sqrt{(z - z_k)^T U^T C U (z - z_k)} + K_{\text{md},k}\sigma_k + b_k \\
=&K_{\text{fa},k}\sqrt{(z - z_k)^T D^T D (z - z_k)} + K_{\text{md},k}\sigma_k + b_k.
\end{aligned} \tag{S-15}$$

Continuing with the substitution of the conversion variable y_k from Eq. (S-10) into the preceding equation yields

$$t_k\sqrt{(x - a_k)^T(x - a_k)} + y_k = t_k\|x - a_k\|_2 + y_k. \tag{S-16}$$

Upon Substituting Eq. (S-6) into the left-hand side of the second constraint in Eq. (12) and transposing the right-hand side to the left, we obtain

$$\begin{aligned}
& s_3^{(\text{opt})} C s_3^{(\text{opt})T} - \sigma_{\text{req},V}^2 \\
=& (s_3^{(\text{full})} + (Uz)^T)C(s_3^{(\text{full})} + (Uz)^T)^T - \sigma_{\text{req},V}^2 \\
=& s_3^{(\text{full})} C s_3^{(\text{full})T} + s_3^{(\text{full})} C U z + (Uz)^T C s_3^{(\text{full})T} + (Uz)^T C U z - \sigma_{\text{req},V}^2.
\end{aligned} \tag{S-17}$$

Expanding each of the first four terms of the preceding formula individually yields

$$\begin{cases}
s_3^{(\text{full})} C s_3^{(\text{full})T} & = e_3^T(G_0^T W_0 G_0)^{-1} G_0^T W_0 W_0^{-1} G[(G_0^T W_0 G_0)^{-1}]^T e_3 = e_3^T(G_0^T W_0 G_0)^{-1} e_3 = \sigma_{\text{best},V}^2 \\
s_3^{(\text{full})} C U z & = e_3^T(G_0^T W_0 G_0)^{-1} G_0^T W_0 W_0^{-1} U z = e_3^T(G_0^T W_0 G_0)^{-1} G_0^T U z = 0 \\
(Uz)^T C s_3^{(\text{full})T} & = z^T U^T W_0^{-1} W_0^T G[(G_0^T W_0 G_0)^{-1}]^T e_3 = z^T U^T G[(G_0^T W_0 G_0)^{-1}]^T e_3 = 0 \\
(Uz)^T C U z & = z^T U^T C U z = z^T D^T D z = x^T x.
\end{cases} \tag{S-18}$$

The aforementioned four equations facilitate the derivation of the subsequent changes in the constraint terms upon altering the decision variables

$$x^T x - (\sigma_{\text{req},V}^2 - \sigma_{\text{best},V}^2) \leq 0. \tag{S-19}$$

The equality constraint can be obtained by a simple transformation. Based on the above, the final expression form of Eq. (S-11) is obtained.

5. Precise penalty function theorem

Theorem 1 Let (x^*, y^*, t^*, u^*) be the global optimal solution of Eq. (17), then the following conclusions hold:

(a)If $u^* \leq M$, then (x^*, y^*, t^*, u^*) is also the global optimal solution of Eq. (20), and $E(x, y, t, u; M, \rho) = 0$ for any $\rho > 0$.

(b)If for some $\rho > 0$, $(\overline{x}, \overline{y}, \overline{t}, \overline{u})$ is the global optimal solution of Eq. (20), and $M = t^*$, then $(\overline{x}, \overline{y}, \overline{t}, \overline{u})$ is also the global optimal solution of Eq. (18).

Theorem 2 Let (x^*, y^*, t^*, u^*) be the global optimal solution of Eq. (18). For some M and $\rho > 0$, let $(x_M^*, y_M^*, t_M^*, u_M^*)$ be the global optimal solution of Eq. (20). Then the following conclusions hold:

(a)If $E(x_M^*, y_M^*, t_M^*, u_M^*; M, \rho) = 0$, and $(x_M^*, y_M^*, t_M^*, u_M^*)$ is a feasible point of Eq. (18), then $u^* \leq u_M^* \leq M$.

(b)If $E(x_M^*, y_M^*, t_M^*, u_M^*; M, \rho) > 0$, and $(x_M^*, y_M^*, t_M^*, u_M^*)$ is not a feasible point of Eq. (18), then $M < u^*$, $u_M^* < u^*$.

(c)If $E(x_M^*, y_M^*, t_M^*, u_M^*; M, \rho) > 0$, and $(x_M^*, y_M^*, t_M^*, u_M^*)$ is a feasible point of Eq. (18), then $(x_M^*, y_M^*, t_M^*, u_M^*)$ is also the global optimal solution of Eq. (18).

Theorem 3 Let $u^* = \min u$, and let $\{(x_i, y_i, t_i, u_i)\}$ denote the sequence generated by the MUBO algorithm. We then draw the following conclusions:

(a)If $\{(x_i, y_i, t_i, u_i)\}$ is a finite sequence, meaning the MUBO algorithm terminates after the $\tilde{i}$-th iteration, then $(x_{\tilde{i}}, y_{\tilde{i}}, t_{\tilde{i}}, u_{\tilde{i}})$ represents the global optimal solution to Eq. (18).

(b)If $\{(x_i, y_i, t_i, u_i)\}$ is an infinite sequence, then the sequence $\{(x_i, y_i, t_i, u_i)\}$ is bounded, and every accumulation point of $\{(x_i, y_i, t_i, u_i)\}$ is a global optimal solution to Eq. (18). The conclusions of Theorem 1, Theorem 2, and Theorem 3 are proved in reference(3, 4).

6. Supplementary results of the BeiDou/GNSS experiment

This section gives the results of availability, VPL, EMT, and accuracy distributions for BeiDou/GNSS experiment of the main text. Fig. S1 gives the results of availability and VPL distribution for Baseline-ARAIM and Optimal-ARAIM. Fig. S2 gives the results of EMT and accuracy distributions for Baseline-ARAIM and Optimal-ARAIM.

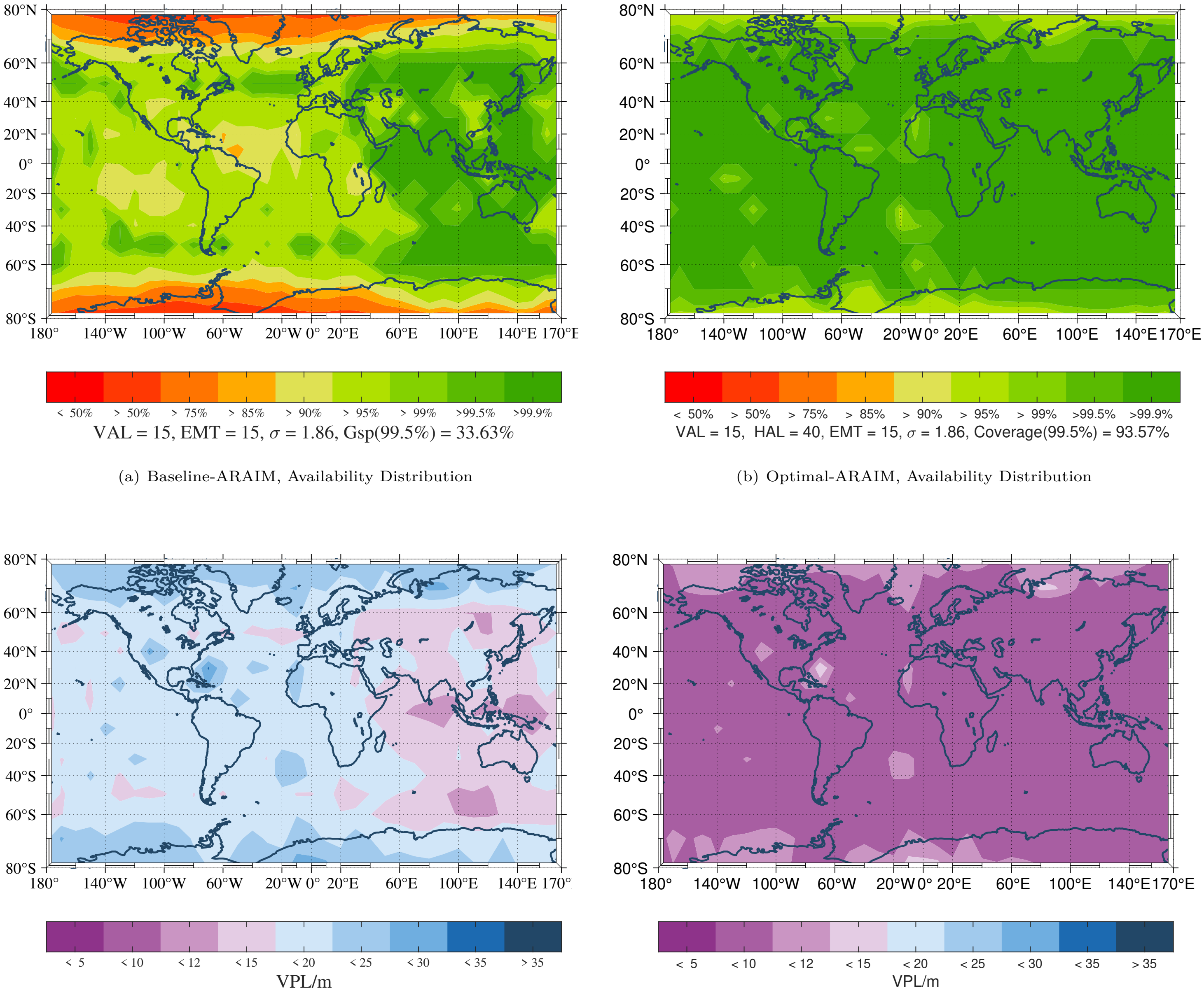

(a) Baseline-ARAIM, Availability Distribution

(b) Optimal-ARAIM, Availability Distribution

(c) Baseline-ARAIM, VPL Distribution

(d) Optimal-ARAIM, VPL Distribution

Fig. S1. Global almanac availability and VPL forecast results

As shown in Fig. S1: (a) Areas with availability greater than 99.5% are within 60°E-170°E and 60°S-60°N, 70°S-80°S and

70°N-80°N have less than 50%, and the remaining areas have availability between 90% and 95%. (b) Availability greater than 99.5% covers most of the region between 60°S-60°N. The rest of the area also remains above 90%. (c) VPL less than 10 m occurs only in the equatorial region from 60°E-170°E, and most of the tested areas are between 15-25 m. (d) The area with VPL less than 10 m covers most of the area between 60°E-170°E and 60°S-60°N. The rest region remains between 10-20 m.

As shown in Fig. S2: The full test areas in (a) and (b) are below 10.5 m, but there are more areas less than 4.5 m in (b) than in (a). (c) and (d) are below 1.86 m for the whole test area, except for some high latitude areas which are larger than 1.86 m. The test area is below 1.86 m for the whole test area. However, (c) has higher accuracy than (d) in some areas, such as in the 20°E-170°E and 60°S-40°N regions, and (c) has more areas with less than 1.2 m than (d).

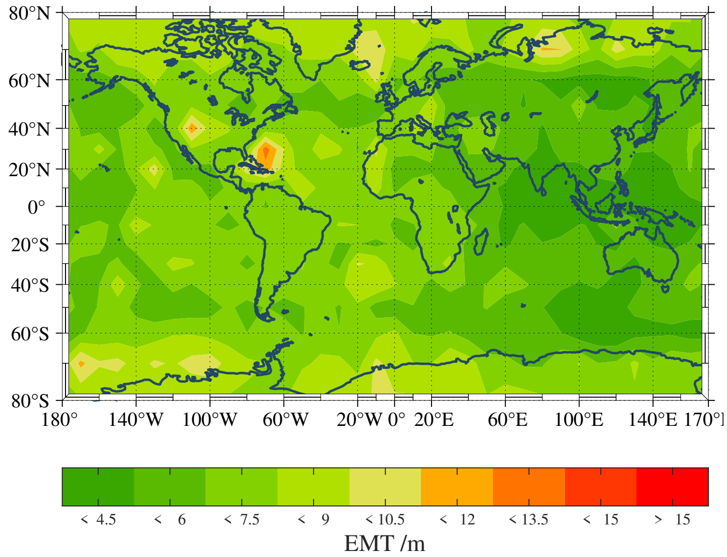

(a) Baseline-ARAIM, EMT Distribution

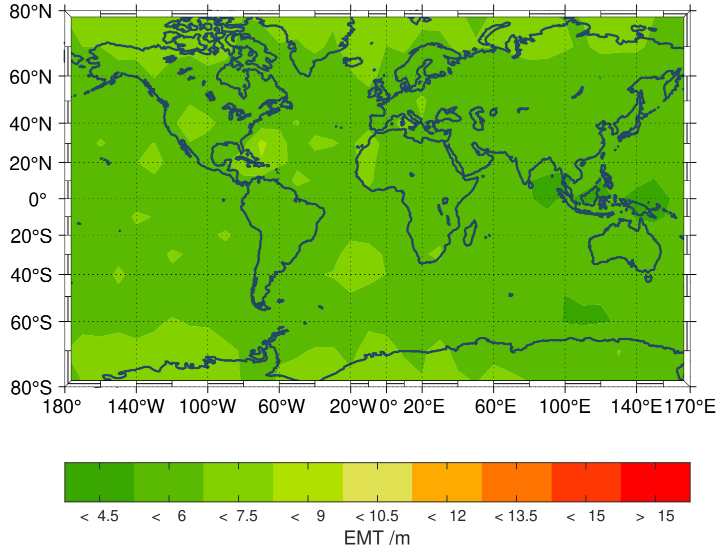

(b) Optimal-ARAIM, EMT Distribution

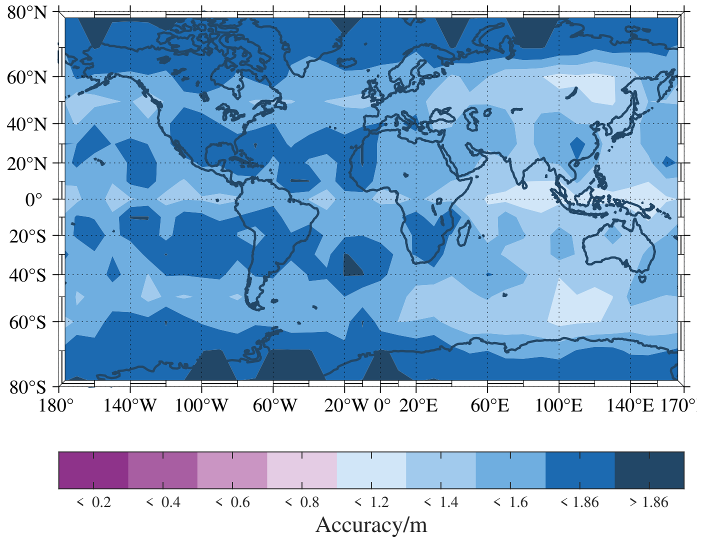

(c) Baseline-ARAIM, Accuracy distribution

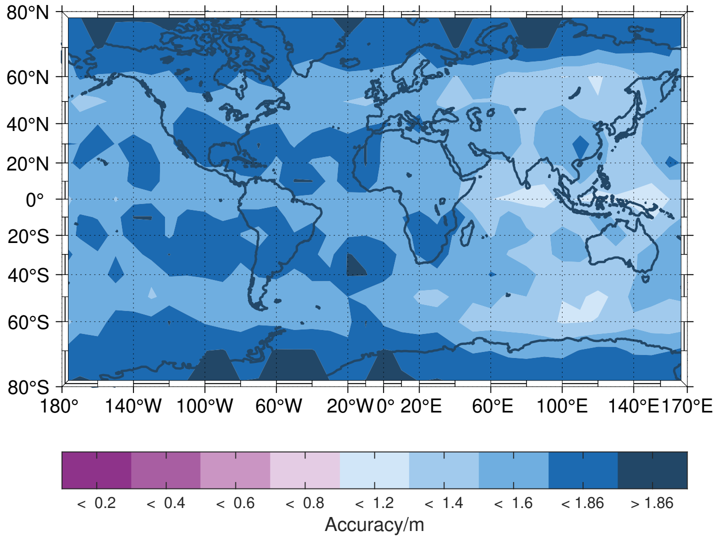

(d) Optimal-ARAIM, Accuracy distribution

Fig. S2. Global almanac EMT and accuracy forecast results

7. Derivation of the maximum order determination equation

A. Specific derivation of Eq. (1)). The probability of a failure-free event for each satellite P_{event,f_k}, i.e., each satellite chooses the normal event $1-P_{\text{event},f_k}$, is thus given by

$$P_0 = \prod_{k=1}^{N}(1-P_{\text{event},k}). \tag{S-20}$$

In the event of a satellite failure, because we cannot determine where the failure occurred and can only assume that every satellite could fail, we has

$$\begin{aligned} P_1 &= \sum_{k=1,\cdots,r} P_{\text{event},f_k} \prod_{k\neq 1,\cdots,r}(1-P_{\text{event},f_k}) \\ &= \prod_{k=1}^{N_{sat}+N_{const}}(1-P_{\text{event},f_k}) \sum_{k=1,\cdots,r}\frac{P_{\text{event},f_k}}{1-P_{\text{event},f_k}} \\ &= P_0 \sum_{k=1,\cdots,r}\frac{P_{\text{event},f_k}}{1-P_{\text{event},f_k}}. \end{aligned} \tag{S-21}$$

Similarly, we can deduce two satellite failures, there:

$$P_2 = P_0 \sum_{k_1<k_2}^{N_{\text{fault}}} \frac{P_{\text{fault}k_1}}{1-P_{\text{fault},k_1}} \frac{P_{\text{fault},k_2}}{1-P_{\text{fault},k_2}}. \tag{S-22}$$

The above is the additional derivation of Eq. (1) of the main text.

B. The upper bound of the probability of r independent faults. If the fault events $f_1, f_2, \cdots, f_r$ occur simultaneously while other independent events remain fault-free, the probability of occurrence is

$$\begin{aligned} P_{f_1,\cdots,f_r} &= \prod_{k=1,\cdots,r} P_{\text{event},f_k} \prod_{k\neq 1,\cdots,r}(1-P_{\text{event},f_k}) \\ &= \prod_{k=1}^{N_{\text{sat}}+N_{\text{const}}}(1-P_{\text{event},f_k}) \prod_{k=1,\cdots,r}\frac{P_{\text{event},f_k}}{1-P_{\text{event},f_k}} \\ &= P_0 \prod_{k=1,\cdots,r}\frac{P_{\text{event},f_k}}{1-P_{\text{event},f_k}}. \end{aligned} \tag{S-23}$$

By observing Eq. (S-23), the first column of the equation of the second multiplication on the left side. and noting that $(1-P_{\text{event},f_k})$ must be less than 1, we can establish that $P_{f_1,\cdots,f_r}$ has a loose upper bound as follows:

$$P_{f_1,\cdots,f_r} < \prod_{k=1,\cdots,r} P_{\text{event},f_k}. \tag{S-24}$$

The probability of one or more faults $(k=1,\cdots,N_{\text{sat}}+N_{\text{const}})$ occurring is the complement of the probability of no faults. Combining this with Eq. Eq. (S-28), we get

$$P_{\text{multiple}}(1,\cdots,N_{\text{sat}}+N_{\text{const}}) = 1-P_0. \tag{S-25}$$

The probability of two or more faults $(k=2,\cdots,N_{\text{sat}}+N_{\text{const}})$ is obtained by subtracting the probability of a single event fault from the probability of one or more faults $\overrightarrow{P_1}$. Thus,we have

$$P_{\text{multiple}}(2,\cdots,N_{\text{sat}}+N_{\text{const}}) = 1-P_0-P_1. \tag{S-26}$$

Similarly, by combining Eq. Eq. (S-25) and Eq. (S-26), the probability of three or more faults $(k=3,\cdots,N_{sat}+N_{const})$ occurring is

$$P_{\text{multiple}}(3,\cdots,N_{\text{sat}}+N_{\text{const}}) = 1-P_0-P_1-P_2. \tag{S-27}$$

Let P_{event,f_k} represent the prior probability of an independent fault event k occurring. The probability of a fault-free event occurring is

$$P_0 = \prod_{k=1}^{N_{\text{sat}}+N_{\text{const}}}(1-P_{\text{event},f_k}). \tag{S-28}$$

Introduce the independent variable $X_k \in \{0,1\}$ satisfying:

$$P(X_k = 1) = P_{\text{event},k}, \quad k = 1, \ldots, N, \qquad S = \sum_{k=1}^{N} X_k, \tag{S-29}$$

wherein X_k is a Bernoulli random variable indicating whether the k-th satellite fails (1 for failure, 0 for healthy). Thus, $P(X_k = 1) = P_{\text{event},k}$ and $P(X_k = 0) = 1 - P_{\text{event},k}$. We are interested in the total number of failures, $S = \sum_{k=1}^{N} X_k$. It is known that the Poisson-Binomial distribution describes the number of successes in N independent but possibly non-identical Bernoulli trials. Its probability generating function is given by:

$$G(s) = \prod_{k=1}^{N} \big[1 - P_{\text{event},k} + P_{\text{event},k}\, s \big]. \tag{S-30}$$

Taking the logarithmic expansion of $G(s)$, we have:

$$G(s) = \exp\big(\sum_{k=1}^{N} \ln\left(1 + P_{\text{event},k}(s-1)\right)\big) \tag{S-31}$$

When each $P_{\text{event},k}$ is very small, the first-order Taylor approximation of the logarithm can be applied:

$$\ln(1 + P_{\text{event},k}(s-1)) \approx P_{\text{event},k}(s-1). \tag{S-32}$$

Thus,

$$G(z) \approx \exp(\sum_{k=1}^{N} P_{\text{event},k}(s-1)) = \exp(u(s-1)), \tag{S-33}$$

where the above expression is the probability generating function of a Poisson distribution with parameter u. Therefore, when $\max_k P_{\text{event},k} \ll 1$ and $u = \sum P_{\text{event},k}$ is not too large, the Poisson distribution with parameter u can be used as an approximation:

$$\begin{cases} P\big(S = r\big) \approx e^{-u}\, \frac{u^r}{r!}, \\ P\big(S \geq r\big) \approx \sum_{i=r}^{N} e^{-u}\, \frac{u^i}{i!}, \\ P\big(S \geq r\big) \approx 1 - \sum_{i=0}^{r-1} e^{-u}\, \frac{u^i}{i!}. \end{cases} \tag{S-34}$$

Based on this approximation, to satisfy $P(S \geq r) < P_{\text{thres}}$, we have:

$$\sum_{i=0}^{r-1} \frac{u^i}{i!} \;\geq\; 1 - P_{\text{thres}}, \tag{S-35}$$

leading to the maximum order determination formula:

$$O_{\text{fault,max}} = \min\Big\{ r \;\Big|\; \sum_{i=0}^{r-1} \frac{u^i}{i!} \geq 1 - P_{\text{thres}} \Big\}. \tag{S-36}$$

The above is a detailed derivation of Eq. (2) in the main text.

C. Reasons for unmonitored probabilities to be subtracted from the PHMI. Denote the raw PHMI (total probability of false alarm in the absence of maximum monitoring order truncation) of the system under all potential fault assumptions as:

$$P_{\text{HMI}} = \sum_{h \in \mathcal{H}} P(h), \tag{S-37}$$

where $\mathcal{H}$ is the set of all possible fault hypotheses. Since the maximum monitoring order $O_{\text{fault,max}}$ is used, only a fraction of the fault hypotheses are monitored. The whole set of hypotheses is divided into a monitored subset $\mathcal{H}_M$ and an unmonitored subset $\mathcal{H}_U$, when

$$\underbrace{\sum_{h \in \mathcal{H}_M} P(h)}_{\text{PHMI}_M} \;+\; \underbrace{\sum_{h \in \mathcal{H}_U} P(h)}_{\text{PHMI}_{NM}} \;=\; P_{\text{HMI}}. \tag{S-38}$$

To compensate for undercounting due to not monitoring higher-order, low-probability faults, we compensate the unmonitored portion as a percentage of the total PHMI so that the final corrected PHMI approximates the original setpoint:

$$P_{\text{HMI,M}} = P_{\text{HMI}} - P_{\text{HMI,NM}} = P_{\text{HMI}}(1 - \frac{P_{\text{HMI,NM}}}{P_{\text{HMI}}}) \tag{S-39}$$

The above is a description of the PHMI calibration (Eq.(3) of the main text).

8. The main text "Results/Evaluations" on BeiDou part of the experimental results added

To extend the experimental results of the Evaluations on the Beidou subsection in the Results section of the main text, the statistics of the number of satellites for the five geostationary stations used are plotted and the accuracy results of Optimal-ARAIM in the Asian regional test are given, as shown in Fig. S3.

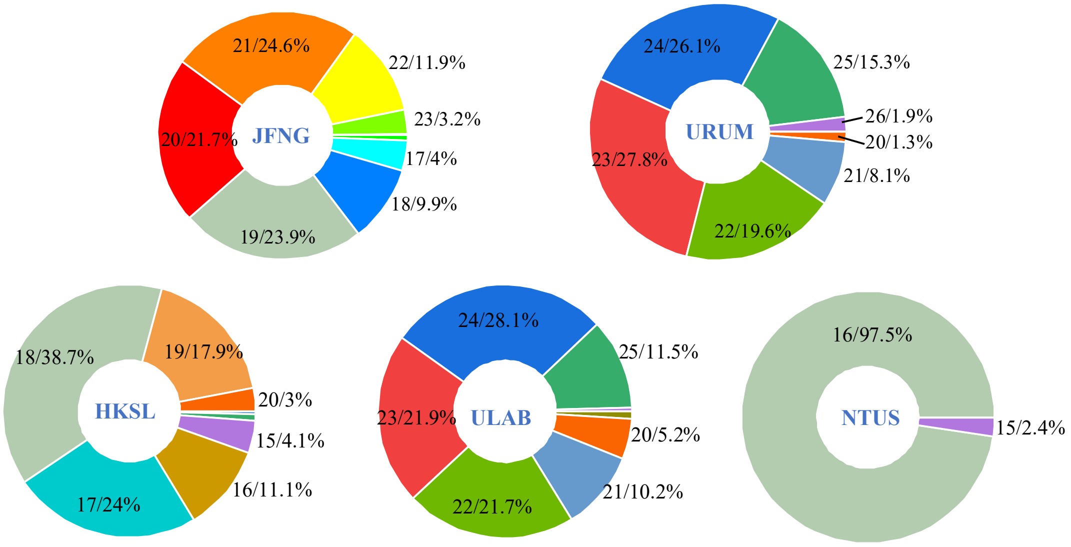

(a) Statistics on the number of satellites (used to supplement Fig. 5 of the main text)

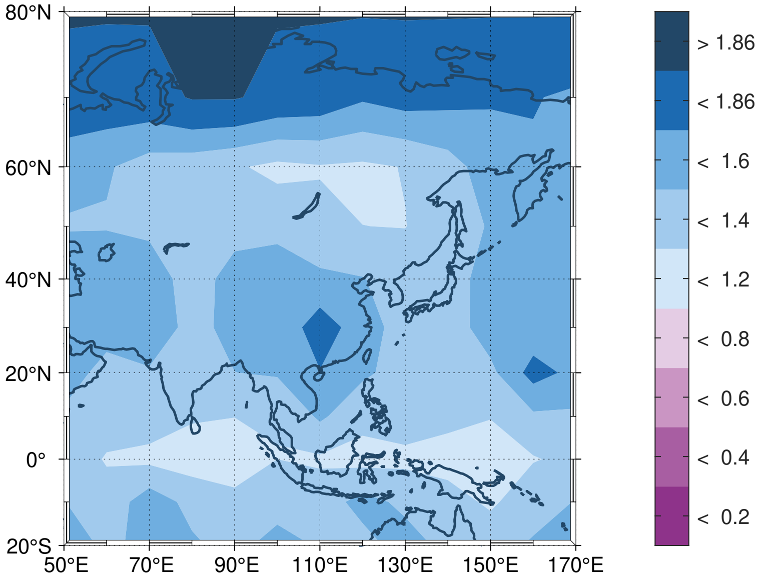

(b) Optimal-ARAIM's accuracy statistics for Asia region (Used to supplement Fig. 7 (a) in the main text)

Fig. S3. Number of satellites and Asia region accuracy distribution

As shown in Fig. S3(a), the number of available satellites for the five stations as a percentage of the total 8,640 calendar elements is represented in the chart. The average number of satellites for the five stations is 15, 22, 17, 23, 20. The number of available satellites at the five stations exceeds 15 during most epochs. This high satellite availability provides a solid basis for evaluating the integrity performance. In the regional accuracy distribution of the optimal-ARAIM shown in Fig. S3(b), the accuracy from 20°S to 80°N latitude remains below 1.86 meters. This indicates that the Optimal-ARAIM method can effectively reduce VPL while satisfying the accuracy requirement.

References

1. J Blanch, et al., Baseline advanced raim user algorithm and possible improvements. *IEEE Transactions on Aerosp. Electron. Syst.* **51**, 713–732 (2015).
2. J Nocedal, SJ Wright, *Numerical optimization.* (Springer), (1999).
3. Z Meng, C Dang, M Jiang, X Xu, R Shen, Exactness and algorithm of an objective penalty function. *J. Glob. Optim.* **56**, 691–711 (2013).
4. J Li, Z Wu, Q Long, A new objective penalty function approach for solving constrained minimax problems. *J. Oper. Res. Soc. China* **2**, 93–108 (2014).
